# Mast cells play role in wound healing through the ZnT2/GPR39/IL-6 axis

**DOI:** 10.1038/s41598-019-47132-5

**Published:** 2019-07-25

**Authors:** Keigo Nishida, Aiko Hasegawa, Satoru Yamasaki, Ryota Uchida, Wakana Ohashi, Yosuke Kurashima, Jun Kunisawa, Shunsuke Kimura, Toshihiko Iwanaga, Hiroshi Watarai, Koji Hase, Hideki Ogura, Manabu Nakayama, Jun-ichi Kashiwakura, Yoshimichi Okayama, Masato Kubo, Osamu Ohara, Hiroshi Kiyono, Haruhiko Koseki, Masaaki Murakami, Toshio Hirano

**Affiliations:** 1 Laboratory of Immune Regulation, Graduate School of Pharmaceutical Sciences, Suzuka University of Medical Science, 3500-3 Minamitamagaki-cho, Suzuka, Mie 513-8670 Japan; 2 Laboratory for Homeostatic Network, RIKEN Center for Integrative Medical Sciences (IMS), 1-7-22 Suehiro-cho, Tsurumi, Yokohama, Kanagawa 230-0045 Japan; 3 Laboratory for Immunotherapy, RIKEN Center for Integrative Medical Sciences (IMS), 1-7-22 Suehiro-cho, Tsurumi, Yokohama, Kanagawa 230-0045 Japan; 4 Laboratory for Cytokine Regulation, RIKEN Center for Integrative Medical Sciences (IMS), 1-7-22 Suehiro-cho, Tsurumi, Yokohama, Kanagawa 230-0045 Japan; 5 Laboratory for Integrative Genomics, RIKEN Center for Integrative Medical Sciences (IMS), 1-7-22 Suehiro-cho, Tsurumi, Yokohama, Kanagawa 230-0045 Japan; 6 Laboratory for Developmental Genetics, RIKEN Center for Integrative Medical Sciences (IMS), 1-7-22 Suehiro-cho, Tsurumi, Yokohama, Kanagawa 230-0045 Japan; 7 Department of Pediatrics, Shinshu University School of Medicine, 3-1-1 Asahi, Matsumoto, Nagano 390-8621 Japan; 8 Department of Molecular and Medical Pharmacology, Graduate School of Medicine and Pharmaceutical Sciences for Research, University of Toyama, Toyama, 930-0194 Japan; 9 Department of Innovative Medicine, Graduate School of Medicine, Chiba University, 1-8-1 Inohana, Chuo-ku, Chiba, 260-8670 Japan; 10 Division of Mucosal Immunology, IMSUT Distinguished Professor Unit, the Institute of Medical Science, The University of Tokyo, 4-6-1 Shirokanedai, Minato-ku, Tokyo, Japan; 11 Department of Mucosal Immunology, Graduate School of Medicine, Chiba University, 1-8-1 Inohana, Chuo-ku, Chiba 260-8670 Japan; 12 Division of Clinical Vaccinology, International Research and Development Center for Mucosal Vaccine, The Institute of Medical Science, The University of Tokyo, 4-6-1 Shirokanedai, Minato-ku, Tokyo, Japan; 13 Institute for Global Prominent Research, Chiba University, 1-8-1 Inohana, Chuo-ku, Chiba, 260-8670 Japan; 14 Chiba University-UC San Diego Center for Mucosal Immunology, Allergy and Vaccines (CU-UCSD cMAV), University of California San Diego, 9500 Gilman Dr. MC 0063, San Diego, CA, 92093-0063 United States; 15 Laboratory of Vaccine Materials, Center for Vaccine and Adjuvant Research, and Laboratory of Gut Environmental System, National Institutes of Biomedical Innovation, Health and Nutrition (NIBIOHN), 7-6-8 Asagi Saito, Ibaraki, Osaka, 567-0085 Japan; 16 Division of Mucosal Vaccines, International Research and Development Center for Mucosal Vaccines, The Institute of Medical Science, The University of Tokyo, 4-6-1 Shirokanedai, Minato-ku, Tokyo 108-8639 Japan; 17 Laboratory of Histology and Cytology, Department of Anatomy, Hokkaido University Graduate School of Medicine, Sapporo, 060-8638 Japan; 18 Department of Immunology and Stem Cell Biology, Faculty of Medicine, Institute of Medical, Pharmaceutical and Health Sciences, Kanazawa University, 13-1 Takaramachi, Kanazawa, Ishikawa 920-8640 Japan; 19 Division of Biochemistry, Faculty of Pharmacy, Keio University, Tokyo, 105-8512 Japan; 20 International Research and Development Center for Mucosal Vaccine, The Institute of Medical Science, The University of Tokyo (IMSUT), 108-8639 Tokyo, Japan; 21 Department of Microbiology, Hyogo College of Medicine 1-1, Mukogawa-cho, Nishinomiya 663-8501 Japan; 22 Laboratory of Medical Omics Research, Department of Frontier Research and Development, Kazusa DNA Research Institute,2-6-7 Kazusa-Kamatari, Kisarazu, Chiba 292-0818 Japan; 23 Laboratory of Immunology, Graduate School of Pharmaceutical Sciences, Hokkaido University, Sapporo, 060-0812 Japan; 24 Allergy and Immunology Project Team, Center for Allergy, Center for Medical Education, Nihon University School of Medicine, 30-1 Oyaguchi Kamicho Itabashi-Ku, Tokyo, 173-8610 Japan; 25 Division of Molecular Pathology, Research Institute for Biomedical Science, Tokyo University of Science, 2669 Yamazaki, Noda-shi, Chiba 278-0022 Japan; 26 Division of Molecular Psychoimmunology, Institute for Genetic Medicine and Graduate School of Medicine, Hokkaido University, Sapporo, 060-815 Japan; 27 Headquarters, National Institutes for Quantum and Radiological Science and Technology, 4-9-1 Anagawa, Inage-ku, Chiba 263-8555 Japan

**Keywords:** Cell biology, Immunology, Immunology, Cell biology, Cell biology

## Abstract

Zinc (Zn) is an essential nutrient and its deficiency causes immunodeficiency and skin disorders. Various cells including mast cells release Zn-containing granules when activated; however, the biological role of the released Zn is currently unclear. Here we report our findings that Zn transporter ZnT2 is required for the release of Zn from mast cells. In addition, we found that Zn and mast cells induce IL-6 production from inflammatory cells such as skin fibroblasts and promote wound healing, a process that involves inflammation. Zn induces the production of a variety of pro-inflammatory cytokines including IL-6 through signaling pathways mediated by the Zn receptor GPR39. Consistent with these findings, wound healing was impaired in mice lacking IL-6 or GPR39. Thus, our results show that Zn and mast cells play a critical role in wound healing through activation of the GPR39/IL-6 signaling axis.

## Introduction

Zinc (Zn) is an essential nutrient, and its deficiency causes growth retardation, immunodeficiency, and skin disorders^1–4^. Zn is critically involved in various physiological functions including development, immunity and wound healing^5–9^, and impaired wound healing can be reversed by Zn supplementation^10,11^. Indeed, many metalloenzymes and transcription factors require Zn to execute their functions^12^. Zn also acts as an intracellular signaling molecule, and changes in its intracellular concentration in response to extracellular stimuli affect a variety of signaling pathways^13–19^. It has been reported that Zn also functions as a neurotransmitter^20,21^. Zn released from activated neurons appears to regulate the overall excitability of the brain through its effects on glutamate and γ-aminobutyric acid (GABA) receptors, and it is also considered important for synaptic plasticity^22,23^. It has also been reported that mast cell granules are rich in Zn and they release Zn like neurotransmitter^24–26^. These reports suggest that Zn in mast cell granules may be involved in mast cell-dependent inflammatory or allergic responses. However, it is currently unclear how Zn released from mast cell regulates inflammatory responses such as those involved in wound repair.

Recent studies have shown that Zn is an endogenous agonist for GPR39, an orphan receptor that is structurally and functionally related to G-protein-coupled receptors^27^. GPR39 is ubiquitously expressed in peripheral tissues including the skin, gut and brain^28–30^. In addition, accumulating *in vitro* evidence suggests that GPR39 mediates Zn-dependent signaling in keratinocytes, colonocytes and neurons^31^. Therefore, GPR39 may function as a physiological receptor for Zn released from several different types of cells.

To determine whether Zn released from mast cells plays an important role in inflammatory processes such as those involved in wound healing, and to explore the mechanism by which Zn functions in these processes, we used genetically engineered mice that have mast cells defective in localizing Zn to granules. The homeostasis of cellular Zn is regulated by two major families of mammalian Zn transporters: the Zip family that increases intracellular Zn, and the ZnT family that extrudes Zn from the cytoplasm either directly into the extracellular environment or into intracellular secretory vesicles. The ZnT family has nine known members^32–36^. The accumulation of Zn in cellular organelles such as granules depends on the members of the ZnT family^37^. For example, ZnT3 is essential for the accumulation of Zn in synaptic vesicles of the neuron^38^. Therefore, we set out to identify the ZnT family member most closely associated with mast cell granules, and examined its effect on Zn accumulation in these granules by generating mutant mice containing a deletion in the relevant ZnT family member.

In this study, we identified ZnT2 as the ZnT family member responsible for Zn accumulation in mast cell granules by using *ZnT2*^−/−^ mice. We also showed that Zn and mast cells play a role in wound healing by inducing IL-6 production via activation of GPR39 at inflammatory sites of several types of cells. Furthermore, we obtained genetic evidence that both IL-6 and GPR39 are required for cutaneous wound healing. Collectively, our findings indicate that Zn is a pro-inflammatory signal and that the Zn/GPR39/IL-6 axis plays a critical role in wound healing.

## Results

### ZnT2 is required for the release of Zn from mast cells

**Figure 1:**
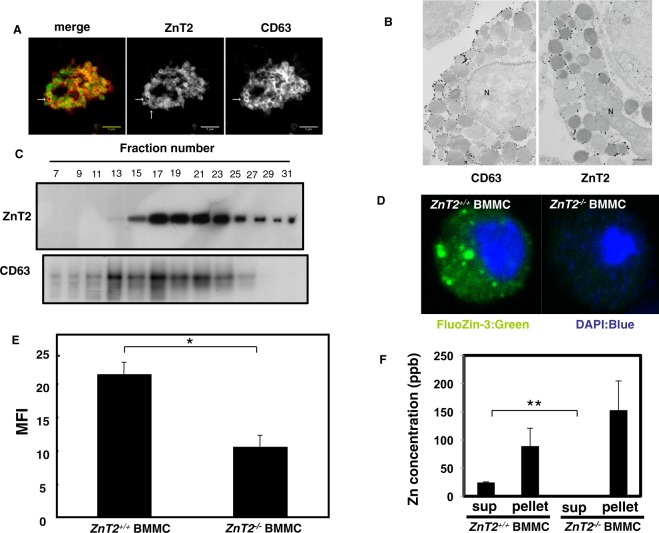
ZnT2 is required for Zn release from stimulated mast cells. (**A**) Double immunostaining of CD63 (red) and ZnT2 (green) in mast cells. ZnT2 is clearly localized at the periphery of granules indicated by an arrow. Scale bars: 5 μm (**B**) Gold particles showing the immunoreactivities for CD63 and ZnT2 are distributed mainly along the membrane of granules. N: nucleus, scale bar: 1 μm (**C**) Nuclear-free cell extract from BMMCs was fractionated by centrifugation in a 0.4–2.0 M sucrose gradient. Proteins in each fraction were analyzed by immunoblotting using anti-ZnT2 and -CD63 antibodies. (**D**) Confocal microscopy of intracellular granule-resident Zn using the Zn indicator FluoZin-3 (green) in BMMCs. Nuclei were stained with DAPI (blue). (**E**) FACS analysis of intracellular granule-resident Zn using FluoZin-3 in BMMCs. The mean fluorescence intensity (MFI) is shown. Values represent the mean + SD. *P < 0.05 (two-tailed Student’s t-test). (**F**) *ZnT2*^+/+^ and *ZnT2*^−/−^ BMMCs were stimulated with 1 μM ionomycin for 30 min. Zn released into the culture supernatant was quantified by ICP-AES. Values represent the mean + SD. **P < 0.01 (two-tailed Student’s t-test). A representative dataset from two (for c, f) or three (for e) experiments, each of which gave similar results, is shown.

To investigate the role of Zn in mast cell granules, we first sought to identify the Zn transporter(s) that transport Zn into this organelle. We examined the expression level of each ZnT family member in bone marrow-derived mast cells (BMMCs) by querying the Reference Database of Immune Cells (ReFDIC)^39^, and found that *ZnT2* is among the highly expressed *ZnTs* in BMMCs (Supplemental Fig. 1). Next, we examined the subcellular localization of ZnT2 in BMMCs by confocal microscopy, and detected the transporter in the cytoplasm, colocalized with a granule marker CD63 (Fig. 1A and Supplemental Fig. 2). To confirm this result, we performed the electron microscopic observation of mast cells with anti-CD63 (granule marker) and anti-ZnT2 antibodies. As shown in Fig. 1B, CD63 and ZnT2 signals were detected around the granule membrane of mast cells. When the BMMC-derived organelles were fractionated by sucrose gradient centrifugation, ZnT2 was mainly detected in CD63-enriched fractions (Fig. 1C and Supplemental Fig. 3). These results identified ZnT2 as a candidate molecule responsible for transporting Zn into mast cell granules.

To clarify the function of ZnT2, we used *ZnT2*^−/−^ mice^40^. BMMCs derived from *ZnT2*^+/−^, but not *ZnT2*^−/−^ bone marrow, expressed *ZnT2* protein, confirming gene inactivation in the mutant (Supplemental Figs 4 and 5). No difference was found in mast cell development between the two genotypes *in vivo* or *in vitro*. As shown in Supplemental Fig. 6A–C, there was no significant difference between the two genotypes in the morphology of skin mast cells, the ratio of peritoneal mast cells as well as the expression of c-kit and FcεRI on the surface of cultured mast cells. We next investigated the Zn content in BMMC-derived mast cell granules from *ZnT2*^−/−^ and WT mice by confocal microscopy using a Zn probe FluoZin-3. FluoZin-3 signal was clearly detected in granule-like structures in the cytoplasm of control BMMCs but not in *ZnT2*^−/−^ BMMCs (Fig. 1D). Consistent with this observation, the intensity of FluoZin-3 signal was also significantly reduced in the *ZnT2*^−/−^ mast cells as detected by flow cytometry (Fig. 1E). As these findings indicated that ZnT2 is important for the accumulation of Zn in mast cell granules, we investigated whether Zn can be released from *ZnT2*^−/−^ BMMCs by inductively coupled plasma-atomic emission spectroscopy (ICP-AES) analysis, and found that Zn was not released following ionomycin stimulation (Fig. 1F). To determine whether this defect altered mast cell function, we examined the ability of *ZnT2*^−/−^ BMMCs to degranulate, synthesize and produce cytokines. *ZnT2*^−/−^ BMMCs released similar amounts of β-hexosaminidase to WT BMMCs and produced comparable amounts of IL-6 and TNFα in response to FcεRI or LPS stimulation (Supplemental Fig. 7A–C), indicating that ZnT2 has little influence on the secretion mechanisms involved in the release of β-hexosaminidase, IL-6 or TNFα in BMMCs.

### Mast cell ZnT2 is required for normal wound healing

**Figure 2:**
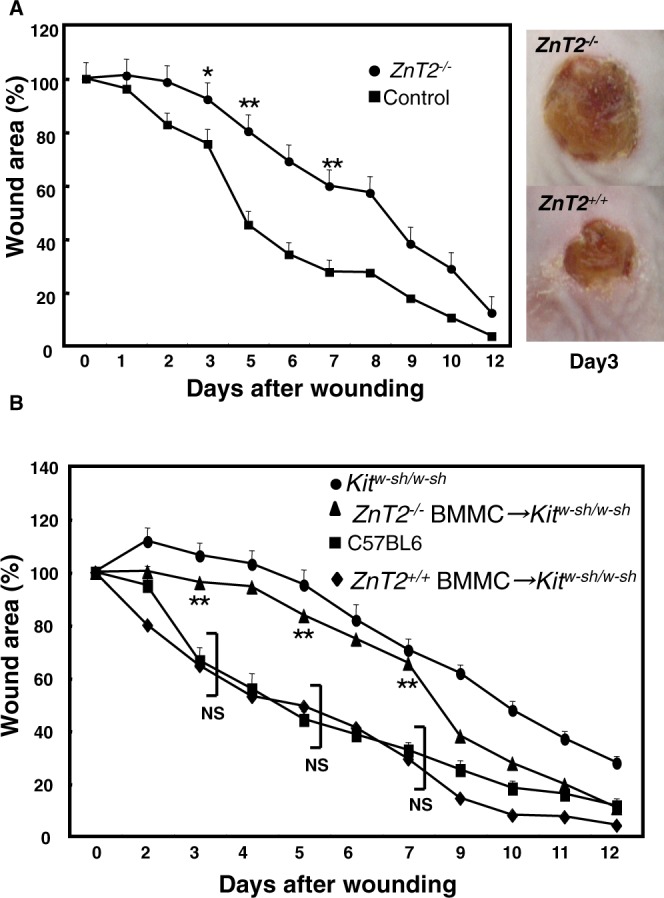
ZnT2 is important for normal wound healing. (**A**) Wound repair in *ZnT2*^−/−^ and control mice. Changes in the wound area over time were expressed as a percentage of the original wound area. Values represent the mean + SEM. Filled circles, *ZnT2*^−/−^ (n = 11 animals; female = 7, male = 4); filled squares, control (n = 12; female = 7, male = 5). *P < 0.05, **P < 0.01 (two-tailed Student’s t-test), *ZnT2*^−/−^ compared with control mice. Photographs show the macroscopic changes in skin wounds 3 days after wounding in *ZnT2*^−/−^ and control mice. (**B**) Wound closure in *Kit*^*W*-*sh*^/*Kit*^*W*-*sh*^ mice (n = 5 animals; female = 2, male = 3) whose dermis was previously reconstituted with control (n = 16 animals; female = 8, male = 8) or *ZnT2*^−/−^ (n = 13; female = 6, male = 7) BMMCs. Data were pooled from at least 3 independent experiments. Values represent the mean + SEM; **P < 0.01 comparing C57BL/6 and *ZnT2*^−/−^ mast cell-reconstituted *Kit*^*W*-*sh*^/*Kit*^*W*-*sh*^ mice (*ZnT2*^−/−^ BMMCs → *Kit*^*W*-*sh*^/*Kit*^*W*-*sh*^ mice); NS P > 0.05 comparing C57BL/6 and *ZnT2*^+/+^ mast cell-reconstituted *Kit*^*W*-*sh*^/*Kit*^*W*-*sh*^ mice (*ZnT2*^+/+^ BMMCs → *Kit*^*W*-*sh*^/*Kit*^*W*-*sh*^ mice).

Given the defective Zn release from *ZnT2*^−/−^ BMMCs, we next examined the role of Zn localized in intracellular granules in a mast cell-mediated inflammatory process using mutant mice. Wound healing is a highly ordered and well-coordinated process involving inflammation, cell proliferation, matrix deposition and tissue remodeling^41^. We and others have shown that mast cells are involved in this process using mast cell-deficient mice such as *Kit*^*W*-*sh*/*W*-*sh*^ mice and MasTRECK mice (Supplemental Fig. 8A,B). To evaluate the role of ZnT2 in mast cell granules in wound healing, *ZnT2*^−/−^ and control mice were subjected to full-thickness excision of shaved dorsal skin and wounds were monitored for up to 12 days. In control mice, the wound area was reduced to 30% of the original area 7 days after injury. However, *ZnT2*^−/−^ mice exhibited impaired wound closure, and the wound remained at 60% of the original area 7 days after injury (Fig. 2A).

To determine whether defective wound healing in *ZnT2*^−/−^ mice was caused by ZnT2 deficiency in mast cells and not by factors derived from other cells or tissues in the wound environment, we engrafted mast cells from control or *ZnT2*^−/−^ mice into *Kit*^*W*-*sh*/*W*-*sh*^ mice, in which mast cells are not observed. The number of mast cells per mm^2^ in the dermis from the back skin in both groups of mice was comparable (Supplemental Fig. 9). The engraftment of BMMCs from control mice, but not *ZnT2*^−/−^ mice, restored wound healing in the *Kit*^*W*-*sh*/*W*-*sh*^ mice (Fig. 2B). Collectively, these findings demonstrate that the expression of ZnT2 in mast cells is required in the early stage of normal wound healing, although we do not completely exclude the possible involvement of ZnT2 expressed by other cells or tissues.

### ZnT2 regulates IL-6 production during wound healing

**Figure 3:**
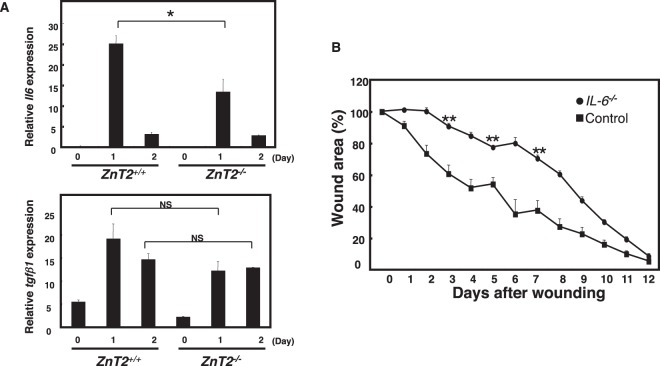
ZnT2 is involved in *Il*-*6* gene induction during wound healing. (**A**) *Il*-*6* and *Tgf*-β*1* mRNA expression in 6 mm of skin surrounding the wound edge 0, 1, and 2 days after injury, quantified by real-time PCR. (**B**) Wound repair was evaluated in *Il*-*6*^−/−^ and control mice. Values represent the mean + SEM. Filled circles, *Il*-*6*^−/−^ (n = 9 animals; female = 5, male = 4); filled squares, control. (n = 9 animals; female = 5, male = 4). **P < 0.01 (two-tailed Student’s t-test), *Il*-*6*^−/−^ compared with control mice.

Wound healing is characterized by three sequential phases: inflammation, proliferation and remodeling^42^. These phases are regulated by various cytokines, including IL-6 and TGF-β. It has been suggested that mast cells are involved in all three phases^43^. We first examined whether ZnT2 was involved in the inflammation phase by making an aseptic, full-thickness incision on the back of control and *ZnT2*^−/−^ mice, as previously described^44^. The expression of *Il*-*6* and *Tgf*-β1 mRNA was higher at the wound edge than in the intact skin of unwounded control mice (Fig. 3A). However, the mRNA expression of *Il*-*6*, but not *Tgf*-β1, was significantly reduced in the wounded *ZnT2*^−/−^ mice compared to wounded controls (Fig. 3A). This result suggested that *il6* mRNA expression after skin injury is regulated by Zn released via ZnT2. To confirm that IL-6 is required for wound healing, we performed the wound-healing assay on *Il*-*6*^−/−^ mice. Wound closure was impaired in the absence of IL-6 in mice 2 to 11 days after injury. The wound area was 70% of the original 7 days after injury, and was consistently larger in the *Il*-*6*^−/−^ than the control mice from 2 to 11 days after injury (Fig. 3B). These observations raised the possibility that Zn derived from mast cells induced inflammatory cytokines such as IL-6 to promote cutaneous wound healing.

### Zn can induce *Il*-*6* gene expression through a Zn receptor GPR39

**Figure 4:**
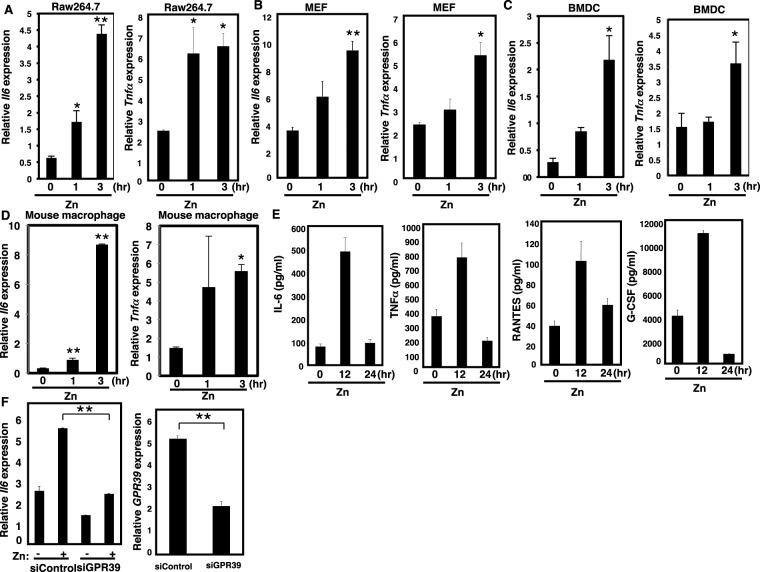
Zn can induce cytokine gene expression through GPR39. Zn-induced *Il*-*6* and *Tnf*α gene expression in (**A**) Raw264.7 cells, (**B**) MEFs, (**C**) BMDCs and (**D**) mouse macrophage. Values represent the mean + SD. (**E**) Zn-induced the expression of IL-6, TNFα, RANTES, and G-CSF protein in Raw264.7 cells, measured by the Bio-Plex suspension array system. (**F**) MEFs were transfected with si*Gpr39*- or si-non-targeting control constructs, and cytokine or *Gpr39* gene expression was measured by real-time PCR. *P < 0.05, **P < 0.01 (Tukey-Kramer multiple comparison test or two-tailed Student’s t-test. A representative dataset from two (for d, e) or three (for a, b, c, f) experiments, each of which gave similar results, is shown.

To test whether Zn can induce the expression of cytokines in immune cells, we measured cytokine gene induction in Raw264.7 macrophage cell lines, mouse embryonic fibroblasts (MEFs), bone marrow-derived dendritic cells (BMDCs), and mouse macrophage treated with 50 μM Zn. We chose this Zn concentration for our experiments based on the previous report^28^. Zn induced not only *Il*-*6* mRNA but also mRNAs for other inflammatory cytokines in all cell types tested (Fig. 4A–D). Consistent with increased mRNA expression, the levels of IL-6, TNFα, RANTES, and G-CSF protein were elevated in Zn-treated Raw264.7 cells (Fig. 4E).

Extracellular Zn has been reported to be a ligand of the GPR39 receptor^45,46^. We therefore examined the role of GPR39 in Zn-dependent cytokine gene expression by gene silencing using siRNA constructs. Zn-triggered cytokine gene induction in MEFs was markedly reduced after GPR39 silencing (Fig. 4F), indicating that the response was mediated by GPR39.

### Zn can regulate the PKC/MAPK/C/EBP signaling cascade

**Figure 5:**
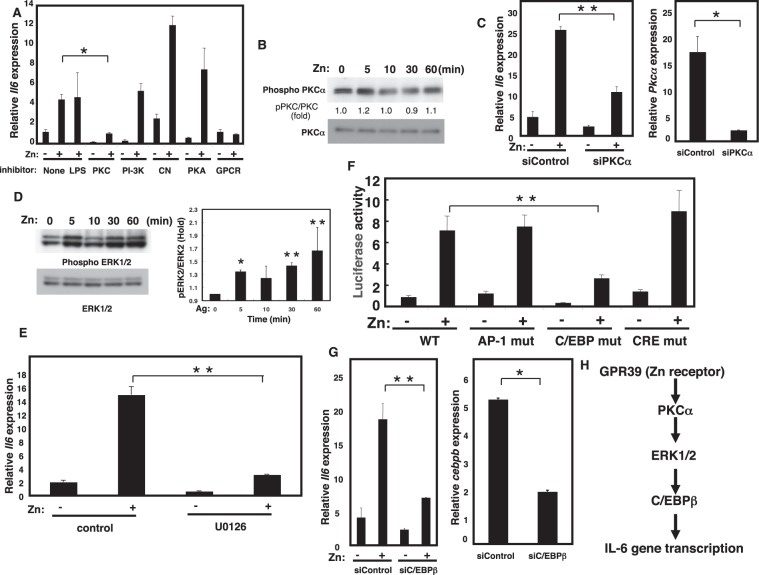
The PKC/MAPK/C/EBP-mediated signaling pathway regulates *Il*-*6* gene induction. (**A**) Raw264.7 cells were treated with 50 μM Zn or 1 μg/ml LPS. Zn**-**induced *Il*-*6* gene expression with PKC inhibitor Gö6976 (10 μM), PI3K inhibitor wortmannin (500 nM), calcineurin inhibitor CsA (2.5 μg/ml), PKA inhibitor 14–22 (1 μM), or GPCR inhibitor GDPβS (1 mM). *Il*-*6* gene induction after Zn stimulation for 3 h was measured by real-time PCR. (**B**) Raw264.7 cells were treated with 50 μM Zn, then PKCα phosphorylation was monitored. Quantification of PKCα phosphorylation normalized to the total PKCα level is shown in the middle. (**C**) MEFs were transfected with si*Pkc*α− or si-non-targeting constructs, and the *Il*-*6* or *Pkc*α gene expression was measured by real-time PCR. (**D**) Raw264.7 cells were treated with 50 μM Zn, then ERK1/2 phosphorylation was monitored. Quantification of ERK2 phosphorylation normalized to the total ERK2 level is shown in the right. *P < 0.05, **P < 0.01 (two-tailed Student’s t-test). (**E**) Raw264.7 cells were treated with 50 μM Zn. Zn**-**induced *Il*-*6* gene induction with MEK inhibitor U0126 (50 μM). *Il*-*6* induction after 3 h of Zn stimulation measured by real-time PCR. (**F**) Raw264.7 cells were stimulated with Zn, then tested for IL-6 reporter activity using the pGL4.21/human *Il*-*6* promoter (−1178/+13) and its mutant constructs (Supplemental Fig. 12). (**G**) MEFs were transfected with si*Cebpb*- or si-non-targeting constructs, and *Il*-*6* or *Cebpb* gene expression was measured by real-time PCR. (**H**) A model for Zn-mediated cytokine gene induction. Zn binds to the Zn receptor GPR39, activating PKC which phosphorylates and activates ERK1/2. Phosphorylated ERK1/2 activates C/EBPβ, which controls the *Il*-*6* gene expression. A representative dataset from two (for b) or three (for a, c, d, f, e, g) experiments, each of which gave similar results, is shown.

We next treated Raw264.7 cells with signaling pathway inhibitors in the presence or absence of Zn. Among the various inhibitors tested, the protein kinase C (PKC) inhibitor Gö6976 and the G-protein inhibitor GDPβS significantly blocked the effect of Zn on cytokine gene expression (Fig. 5A), while the response was not blocked by the protein kinase A (PKA) inhibitor 14–22, the PI-3K inhibitor wortmannin, or the calcineurin inhibitor cyclosporin A (CsA) (Fig. 5A). Based on these results, we tested whether Zn regulates PKC phosphorylation and found that it did (Fig. 5B and Supplemental Fig. 10). siRNA targeting PKC alpha abolished Zn-mediated *Il*-*6* gene induction in MEFs, confirming the involvement of PKC in Zn-mediated cytokine gene induction (Fig. 5C). PKC activation is known to induce the phosphorylation of ERK, and we found that Zn induced ERK phosphorylation, confirming that ERK acts downstream of PKC in this pathway (Fig. 5D and Supplemental Fig. 11). Consistent with this finding, the MAPK inhibitor U0126 significantly reduced Zn-mediated cytokine gene induction (Fig. 5E).

We next examined the molecular mechanism of *Il*-*6* gene regulation. Analysis of *Il*-*6* promoter revealed putative binding sites for several transcription factors including AP1, CRE, and C/EBP (Supplemental Fig. 12). To dissect the transcription factor(s) involved in Zn-mediated cytokine gene induction, we performed a luciferase reporter assay using the *Il*-*6* promoter and constructs containing mutated transcription factor binding sites (Supplemental Fig. 12). Zn-mediated luciferase activity was decreased in the C/EBP mutant (Fig. 5F), suggesting that C/EBP is involved in Zn-mediated cytokine gene induction. *Il*-*6* gene induction was also suppressed in cells transfected with *Cebpb* siRNA (Fig. 5G), confirming the involvement of C/EBP. Taken together, these data are consistent with the mechanism by which Zn activates the GPR39/PKC/ERK/C/EBP signaling pathway and induces cytokine gene expression (Fig. 5H).

### GPR39 is required for normal wound healing

**Figure 6:**
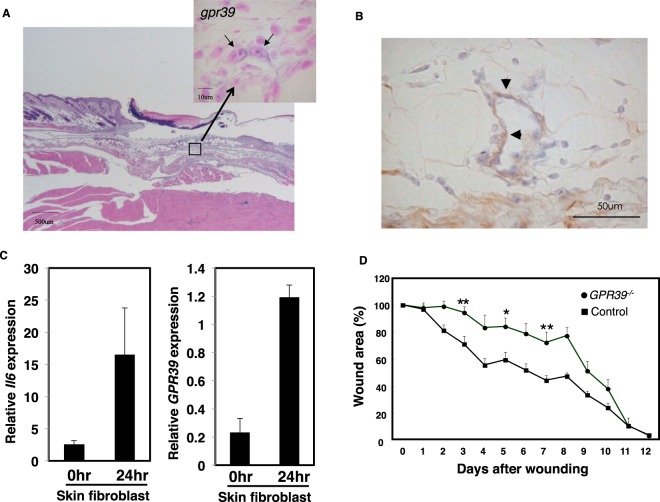
GPR39 is important for normal wound healing. (**A**) *In situ* hybridization analysis of skin wound samples from WT mice 3 days after injury. Skin samples were hybridized with a *Gpr39*-specific probe. Enlarged image of the boxed area is shown (upper right). The bar indicates 10 µm. (**B**) *In situ* hybridization and immunohistochemistry analysis of skin wound samples from WT mice 3 days after injury. Skin samples were hybridized with a *Gpr39*-specific probe. Skin fibroblast was then detected with anti-fibroblasts antibody (clone ER-TR7). The arrowhead indicates *Gpr39* and fibroblast double-positive cell in the skin. The bar indicates 50 µm. (**C**) Real time-PCR analysis of skin fibroblast samples from WT mice 1 days after injury. The expression of *Il*-*6* (left panel) and *Gpr39* (right panel) were examined by real time-PCR analysis. Relative expressions were normalized against *G3pdh*. Data are means ± SD (n = 4 to 6). (**D**) Wound repair was evaluated in *Gpr39*^−/−^ and control mice as in Fig. 2A. Values represent the mean + SEM. Filled circles, *Gpr39*^−/−^ (n = 8 animals; female = 5, male = 3); filled squares, control (n = 10 animals; female = 6, male = 4). *P < 0.05, **P < 0.01 (two-tailed Student’s t-test), *Gpr39*^−/−^ compared with control mice.

To examine the role of GPR39 in wound healing, we first confirmed that *Gpr39* was expressed in the hypodermis under the injury site (Fig. 6A). The *gpr39* signal was not detected in unwounded skin in our *in situ* hybridization experiment (Supplemental Fig. 13). Double *in situ* analysis indicated that *Gpr39* was expressed in fibroblasts (Fig. 6B). Next, we investigated whether *gpr39*-expressed fibroblasts can produce IL-6 upon wound stimulation. We sorted skin fibroblasts from unwounded or wounded skin and checked the expression of IL-6 and GPR39 in skin fibroblasts by qPCR. As shown in Fig. 6C, the expression of IL-6 and GPR39 in the unwounded skin fibroblasts was very low. On the other hand, expression of IL-6 and GPR39 was increased in the wounded skin fibroblasts, suggesting that *gpr39*-expressed fibroblasts can produce IL-6 upon injury (Fig. 6C). Consistent with our observations, *Gpr39*^−/−^ mice showed impaired wound healing at the early stage in normal skin (Fig. 6D).

**Figure 7:**
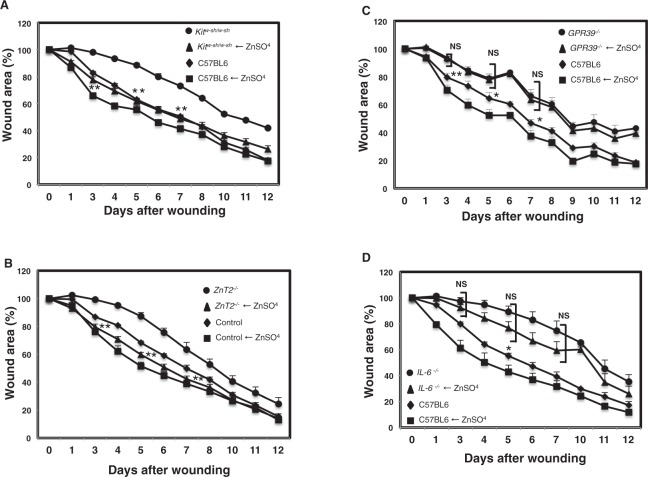
Zn is required for normal wound healing. Wound repair was evaluated in Zn-treated *Kit*^*W*-*sh*^/*Kit*^*W*-*sh*^, *ZnT2*^−/−^, *Gpr39*^−/−^, *Il*-*6*^−/−^, and control mice. Values represent the mean + SEM. (**A**) Wound repair monitored over 12 days in Zn treated-*Kit*^*W*-*sh*^/*Kit*^*W*-*sh*^ mice and control mice. Changes in the wound area in *Kit*^*W*-*sh*^/*Kit*^*W*-*sh*^ (n = 6 animals; female = 2, male = 4, filled circles), Zn-treated *Kit*^*W*-*sh*^/*Kit*^*W*-*sh*^ (n = 6, animals; female = 2, male = 4, filled triangles), control (n = 5, animals; female = 3, male = 2, filled diamonds), Zn-treated control (n = 5, animals; female = 2, male = 3, filled squares). **P < 0.01 (two-tailed Student’s t-test), *Kit*^*W*-*sh*^/*Kit*^*W*-*sh*^ compared with Zn-treated *Kit*^*W*-*sh*^/*Kit*^*W*-*sh*^ mice. (**B**) Wound repair monitored over 12 days in Zn-treated *ZnT2*^−/−^ mice and control mice. Changes in the wound area in *ZnT2*^−/−^ mice (n = 6, animals; female = 2, male = 4, filled circles), Zn-treated *ZnT2*^−/−^ mice (n = 6, animals; female = 3, male = 3, filled triangles), control (n = 5, animals; female = 2, male = 3, filled diamonds), Zn-treated control (n = 6, animals; female = 2, male = 4, filled squares). **P < 0.01 (two-tailed Student’s t-test), *ZnT2*^−/−^ compared with Zn-treated *ZnT2*^−/−^ mice. (**C**) Wound repair monitored over 12 days in Zn treated- *Gpr39*^−/−^ mice and control mice. Changes in the wound area in *Gpr39*^−/−^ (n = 7, animals; female = 5, male = 2, filled circles), Zn-treated *Gpr39*^−/−^ (n = 7, animals; female = 3, male = 4, filled triangles), control (n = 5, animals; female = 2, male = 3, filled diamonds), Zn-treated control (n = 6, animals; female = 3, male = 2, filled squares). N.S., not significant, *P < 0.05, **P < 0.01 (two-tailed Student’s t-test), Statistical significance between Zn-treated *Gpr39*^−/−^ and control mice is shown. (**D**) Wound repair monitored over 12 days in Zn treated- *Il*-*6*^−/−^ mice and control mice. Changes in the wound area in *Il*-*6*^−/−^ (n = 5, animals; female = 2, male = 3, filled circles), Zn-treated *Il*-*6*^−/−^ (n = 6, animals; female = 4, male = 2, filled triangles), control (n = 4, animals; female = 1, male = 3, filled diamonds), Zn-treated control (n = 4, animals; female = 1, male = 3, filled squares). Statistical significance between Zn-treated *Il*-*6*^−/−^ and control mice is shown. N.S., not significant, *P < 0.05.

Finally, we evaluated whether Zn injection into *Kit*^*w*-*sh*/*w*-*sh*^, *ZnT2*^−/−^, *GPR39*^−/−^, and *IL*-*6*^−/−^ mice can restore wound healing deficiency in these mutant mice. As shown in Fig. 7A,B, addition of Zn to the wound area improved the delay in wound healing in *Kit*^*W*-*sh*/*W*-*sh*^ and ZnT2^−/−^ mice compared to their respective untreated controls. However, addition of Zn to the wound area in *GPR39*^−/−^ mice did not rescue the delay in wound healing in *GPR39*^−/−^ mice (Fig. 7C). Furthermore, addition of Zn to the wound area of *Il*-*6*^−/−^ mice rescued the delay in wound healing in *Il*-*6*^−/−^ mice compared to control mice, as shown in Fig. 7D. These results indicated that Zn directly promotes wound repair through Zn receptor GPR39.

Collectively, our findings indicated that Zn and mast cells regulate wound healing through GPR39-mediated IL-6 production by fibroblasts and perhaps other cell types. (Supplemental Fig. 14).

## Discussion

It has long been known that Zn deficiency is associated with poor wound healing^10,11^. This has largely been speculated to be due to the general role of Zn in DNA and protein synthesis^47^. Thus, Zn is known to play an important role in promoting cutaneous wound healing, but its precise mechanism has not been fully understood. In this study, we used a combination of approaches including genetically engineered knockout mice, siRNA gene silencing, and signaling pathway inhibitors to investigate the role of Zn in wound healing and elucidate the mechanism by which it functions. Our findings demonstrate the following: (1) ZnT2 is required for the accumulation and release of Zn from mast cells, (2) Zn activates various types of cells and transmits intracellular signals through GPR39, (3) Zn/GPR39-mediated signaling induces the production of cytokines including the major inflammatory cytokine IL-6^48^ via the PKC/MAPK/C/EBP pathway, and (4) ZnT2, IL-6, and GPR39 are all required in the early stage of normal wound healing. (5) Zinc supplementation can improve the delay in wound healing in mast cell-deficient *Kit*^*w*-*sh*/*w*-*sh*^ mice and zinc transporter-deficient *ZnT2*^−/−^ mice. Thus, our findings revealed a novel role of Zn and mast cells in inflammation. Our results clearly showed that Zn and mast cells are required for production of IL-6 via GPR39 in several different types of cells present at sites of inflammation, and we conclude that mast cells promote wound healing through the Zn/GPR39/IL-6 axis.

We and others have previously shown that IL-6 is required for normal wound healing^49,50^. The wound healing process includes inflammation. During the inflammatory phase, platelet aggregation is followed by infiltration of leukocytes into the wound site. Cell debris and bacteria are phagocytosed and removed from the wound site by white blood cells. The inflammatory phase then transitions to the proliferative phase. We showed that the expression of *Il*-*6* mRNA was significantly reduced in the wounded *ZnT2*^−/−^ mice compared to wounded controls, suggesting IL-6 contributes to the inflammatory phase of wound healing. In addition, our results indicated that Zn receptor GPR39 on skin fibroblasts that receive Zn from mast cells spread IL-6 around the wound site. This idea is supported by the results showing defects in wound healing in *ZnT2*^−/−^, *GPR39*^−/−^, and *IL*-*6*^−/−^ mice. Indeed, it has been reported that GPR39 is involved in skin wound healing^51^. These results suggest that ZnT2-dependent GPR39/IL-6 axis can amplify the cytokine in the inflammatory phase of normal wound healing.

Zn was previously reported as a potential endogenous agonist for GPR39, but the physiological ligand for this receptor has not been identified^45,46^. Our results demonstrate that Zn is indeed a physiological ligand and an agonist for GPR39 at sites of inflammation. Furthermore, we presented evidence that directly link extracellular Zn to intracellular signaling and cytokine production. We showed that Zn can activate the PKC/MAPK/C/EBP signaling cascade leading to *Il*-*6* gene induction. We showed that the G-protein inhibitor GDPβS blocked the effect of Zn on cytokine gene expression, indicating that the Zn receptor GPR39 utilizes G protein to transmit downstream target molecules. Previous reports showing that the activation of GPR39 by Zn stimulates both PLC activity and cAMP production through Gαθ supports this result^31,46^. It is well known that PLC hydrolyzes phosphatidylinositol 4,5-bisphosphate (PIP_2_) to diacylglycerol (DAG) and inositol triphosphate (IP_3_) and that DAG activates PKC. Furthermore, ERK can phosphorylate C/EBP, promoting its nuclear translocation^52^. These reports combined with our observations strongly suggest that the GPR39/PKC/MAPK/C/EBP signaling cascade is required for Zn-mediated cytokine gene induction and this mechanism may share a common role in Zn-dependent inflammatory disorders.

Our studies also revealed the role of ZnT2 in the accumulation of Zn in mast cell granules. Similarly, ZnT3 was shown to be required for the accumulation of Zn in neuronal synaptic vesicles prior to its release as a neurotransmitter^38,53^. The ZnT2 expression is primarily confined to specialized secretory tissues such as the mammary and prostate glands^54^. Recent studies that identified a genetic mutation in *ZnT2* in women producing breast milk with low concentration of Zn that causes severe neonatal Zn deficiency suggested that ZnT2 is essential for the optimal secretion of Zn into milk from the mammary gland^55^. In addition, ZnT8 is required for the accumulation of Zn in the insulin granules in pancreatic β-cells where Zn regulates insulin clearance^56^. Collectively, these reports and the results of our current study demonstrate the critical role of the ZnT family in the accumulation of Zn in cellular organelles as well as the physiological roles of released Zn.

Our findings provide new insights into the molecular mechanisms of wound healing involving mast cells. Our data showing that IL-6 is a major inflammatory cytokine with key roles in autoimmune and inflammatory diseases suggest that the Zn/GPR39/IL-6/cytokine axis may be involved in a variety of inflammatory diseases^48,57^. In addition, many cells have Zn-containing granules and are likely to release it upon stimulation^25,26,58–60^, and cells in inflammatory tissues such as fibroblasts, macrophages, and dendritic cells, express GPR39 as shown in our data. This study paves the way for future research on therapeutic targets in inflammatory diseases for which Zn-containing cells and other inflammatory cells that affect the Zn/GPR39/IL-6/signaling axis are responsible.

## Methods

### Mice

C57BL/6J mice were obtained from CREA Japan. Mast cell-deficient *Kit*^*W*-*sh*/*W*-*sh*^ mice were provided by the RIKEN BioResource Center (RBRC No. RBRC01888) and have been backcrossed at least 10 times to C57BL/6 at RIKEN. *Il*-*6*^−/−^ mice (kindly provided by M. Kopf, Max-Planck-Institute of Immunobiology, Germany, and Y. Iwakura, Tokyo University of Science, Japan) were backcrossed with C57BL/6 mice for >10 generations. *Gpr39*^−/−^ mice were generated by Deltagen (San Mateo, CA, USA)^61^ and backcrossed to a C57BL/6 background for 5 generations. Mast-TRECK mice were provided by Dr. M. Kubo (kindly provided by Tokyo University of Science, Japan). Mas-TRECK tg mice received intraperitoneal injection of 250 ng diphtheria toxin (D0564, Sigma) for 5 consecutive days^62^. Mice used for all experiments were between 6–11 weeks of age. We obtained approval from the Animal Research Committee at RIKEN and the animal research committee at Suzuka University of Medical Science for all animal experiments performed in this study. All methods were performed in accordance with the relevant guidelines and regulations.

### Antibodies and reagents

Anti-ZnT2 antibodies were raised by immunizing rabbits with KLH-conjugated peptide (GKFNFHTMTIQIEKYSEDMKNCQACQGPLE). The rabbits were first injected with 200 μg of KLH-conjugated ZnT2 peptide in complete Freund’s adjuvant and then boosted every week with 50 μg of the antigen in incomplete Freund’s adjuvant. Anti-CD63 (D263-3) antibody was purchased from MBL. Anti-PKCα (SC-208, C-20), anti-phospho-PKC (9371S, S660), anti-GPR39 (ab39227), anti-phospho ERK1/2 (V803A), and anti-ERK1/2 (V114A) antibodies were obtained from Santa Cruz, Cell Signaling, Abcam, and Promega, respectively. Gö6976 (365250), Wortmannin (681675), Cyclosporin A (239835), PKA inhibitor 14–22 (476485), GDPβS (371543), and U0126 (662005) were purchased from Calbiochem. LPS (L2637, LPS from Escherichia coli O55:B5) was purchased from SIGMA.

### Cell lysates and immunoblotting

Cells were harvested, lysed with lysis buffer (20 mM Tris-HCl pH 7.4, 150 mM NaCl, 1% NP-40, proteinase inhibitors, 5 μg/ml pepstatin, 10 μg/ml leupeptin) for 30 min at 4 °C and spun at 12,000 × g, 4 °C for 30 min. The eluted and reduced samples were resolved by SDS-PAGE using a 5–20% gradient polyacrylamide gel (197–15011, Wako), and transferred to a PVDF membrane (IPVH00010, Immobilon-P, Millipore). For immunoblotting, the membranes were incubated with primary antibodies. The membranes were then incubated with HRP-conjugated anti-mouse (62–6520, Thermo Fisher Scientific) or anti-rabbit (65–6120, Thermo Fisher Scientific) antibody for 1 h at room temperature. After extensive washing of the membranes, immunoreactive proteins were visualized using the Western Lightning-ECL system (RPN2232, GE-Healthcare), according to the manufacturer’s instructions. The PVDF membranes were exposed to Fuji RX film (Fuji, RX-U) and densitometric analysis was performed using an LAS-1000 fluorescence image analyzer (Fujifilm, Japan)^18^.

### Wounding and measurement of the wound area

Full-thickness wounds (one per mouse) were induced on the lower back skin of mice as described previously^43^. Mice were used at 6–11 weeks of age, when all the hair follicles on the back skin were in the telogen (resting) phase of the hair cycle. Briefly, the dorsal hair was shaved 24 h before wounding. Mice were anesthetized intraperitoneally with Somnopentyl, and the skin was wiped with 70% ethanol and then wounded using a 6 mm-diameter biopsy punch (BP-60F, Kai Industries, Tokyo, Japan). The wound area was evaluated at various time points after wounding by measuring the diameters along the rostrocaudal and perpendicular (left-right) axes with an ABS Digimatic Caliper (Mitutoyo, Japan) and then calculating the area. The change in wound area was expressed as the percentage of the initial wound area.

### Mast cell engraftment

Mast cell engraftment was prepared as described previously^43^. BMMCs derived from *ZnT2*^−/−^ and *ZnT2*^+/+^ mice were transferred by intradermal injection (10^6^ in 200 µl DMEM per cm^2^, 20 injections of 10 µl each) into an area of 4 cm^2^ on the back skin of 4-week-old *Kit*^*W*-*sh*/*W*-*sh*^ mice. Four weeks after adoptive transfer of BMMCs, mice were used for experiments. To observe the skin mast cells, paraffin sections were fixed and stained with nuclear fast red and Alcian blue or Toluidine blue, and the Alcian blue or Toluidine blue-stained cells in each sample were counted.

### Primary mast cell cultures and cell lines

BMMCs (bone-marrow derived mast cells), BMDCs (bone-marrow derived dendritic cells), and peritoneal macrophages were prepared as described^18,63–65^. For the preparation of BMMCs, bone-marrow cells obtained from 8-week-old C57BL6J or BALB/c mice were cultured in RPMI 1640 supplemented with 10% heat-inactivated FBS, 10 mU/mL penicillin, 0.1 mg/mL streptomycin and the culture supernatant of IL-3-producing CHO cells (CHOmIL-3-3-12M; a gift from T. Sudo, Toray Industry, Kanagawa, Japan), and incubated in a humidified 5% CO_2_ and 95% air at 37 °C. After 4–5 weeks of culturing, cell-surface expression of FcεRI and c-Kit was confirmed and these cells were used for experiments (<95% mast cells). For the preparation of BMDCs, BMDCs were generated from bone marrow cells with RPMI 1640 medium containing 10% FCS in the presence of murine GM-CSF (20 ng/ml, PEPRO TECH, AF-315-03) or the culture supernatant of GM-CSF–producing CHO cells, as described previously^64^. Loosely adherent clustering cells were harvested on days 6–8, and CD11c^+^ DCs were isolated by the IMag Cell Separation System with anti-CD11c mAb-bound beads or FACSAria (BD Biosciences). The sorted CD11c^+^ DCs were immature (CD11c^high^ MHC class II^low^), and the purity was >95%. For the preparation of peritoneal macrophages, C57BL/6J mice received an intraperitoneal (i.p.) injection of 1 ml of 3% (w/v) thioglycollate. Four to five days after injection, the peritoneal macrophages were collected by flushing the peritoneal cavity and were cultured in RPMI medium containing 10% FBS. Raw264.7 cells and MEFs were maintained in RPMI1640 supplemented with 10% FBS, penicillin and streptomycin. MEFs were prepared from C57BL/6J fetuses (13.5 days postcoitus)^66^.

### RNA interference for GPR39, PKCα and C/EBPβ

To knock down GPR39, PKCα, and C/EBPβ, MEFs were transfected with small interfering RNA (siRNA) against GPR39, PKCα, and C/EBPβ or with nontargeting siRNA (SMARTpool; GE-Healthcare) using Lipofectamine RNAiMAX (13778-150, Thermo Fisher Scientific), according to the manufacturer’s instructions. Briefly, MEFs (5 × 10^5^ cell per well) were transfected with siRNA to a final concentration of 200 nM using Lipofectamine RNAiMAX. Forty-eight hours later, cells were stimulated with 50 μM Zn for 3 hrs followed by real-time Quantitative RT-PCR analysis to determine changes in the level of transcription.

### Measurement of cytokines

Raw264.7 cells (0.5 × 10^5^ cells) were treated with 50 μM Zn for 12 and 24 hrs. Cytokines were analyzed by the Bio-Plex suspension array system (Bio-Rad) according to the manufacturer’s protocol.

### Measurement of Zn

Extracts from BMMCs (0.9 × 10^6^ cells) were diluted with water to a total volume of 25 ml and maintained under acidic conditions with nitric acid. Concentrations of Zn^2+^ ions in the diluted extracts were measured by an ICP-AA spectrometer (Shimadzu Sequential Plasma Spectrometer ICPS-8100, Japan) calibrated using calibration curves in the range of 0 to 100 ppb of Zn^2+^.

### Confocal microscopy

BMMCs were allowed to adhere to glass slides (Matsunami, Japan). In some experiments, 10 μM FluoZin-3 (F-24195, Thermo Fisher Scientific) was added for 30 min, and the cells were then fixed with 4% paraformaldehyde in PBS. BMMCs were immunostained after being permeabilized with BD Perm/Wash buffer (554723, BD Biosciences). Antibodies were diluted in buffer containing 1% BSA (anti-ZnT2 at a dilution of 1:25 and anti-CD63 at 1:100). The cells were washed with PBS, then mounted with Mounting Medium (S302380, Dako Cytomation). Confocal microscopy was carried out using the TCS SL system (Leica, Germany). Images were transferred to Adobe Photoshop CS3.

### Electron microscopy

Electron microscopic observation was carried out by the silver-intensified immunogold method. Cryostat sections from the cell pellets were incubated with anti-CD63 or anti-ZnT2 antibodies, followed by an incubation with goat anti-rat IgG or anti-rabbit IgG covalently linked 1-nm gold particles (1:200; Nanoprobes, Yaphank, NK). After silver enhancement using a kit (HQ silver; Nanoprobes), the sections were osmificated, dehydrated, and directly embedded in Epon (Nisshin EM, Tokyo, Japan). Ultrathin sections were prepared and stained with both uranyl acetate and lead citrate for observation under an electron microscope (H-7100; Hitachi, Tokyo, Japan).

### Histological and *in situ* hybridization analyses

Mouse tissue, fixed with G-Fix (Genostaff), was embedded in paraffin on CT-Pro20 (Genostaff) using G-Nox (Genostaff) as a less toxic organic solvent for xylene, and sectioned at 4 μm.

*In situ* hybridization was performed using the ISH Reagent Kit (Genostaff) according to the manufacturer’s instructions. Tissue sections were deparaffinized with G-Nox, and rehydrated through a graded series of ethanol and PBS. The sections were fixed with 10% NBF (10%Formalin in PBS) for 30 min at 37 °C and washed in distilled water, placed in 0.2% HCl for 10 min at 37 °C and washed in PBS, treated with 15 μg/ml Proteinase K (Wako Pure Chemical Industries) in PBS for 10 min at 37 °C and washed in PBS, then placed within a coplin jar containing 1xG-Wash (Genostaff), equal to 1xSSC. Hybridization was performed with probes for *Gpr39* at concentrations of 250 ng/ml in G-Hybo-L (Genostaff) for 16 hr at 60 °C. After hybridization, the sections were washed in 1xG-Wash for 10 min at 60 °C, 50% formamide in 1xG-Wash for 10 min at 60 °C. Then the sections were washed twice in 1xG-Wash for 10 min at 60 °C twice in 0.1xG-Wash for 10 min at 60 °C, and twice in TBST (0.1% Tween20 in TBS) at RT. After treatment with 1xG-Block (Genostaff) for 15 min at RT, the sections were incubated with anti-DIG AP conjugate (Roche Diagnostics) diluted 1:2000 with x50G-Block (Genostaff) in TBST for 1 hr at RT. The sections were washed twice in TBST and then incubated in 100 mM NaCl, 50 mM MgCl_2_, 0.1% Tween20, 100 mM Tris-HCl, pH 9.5. Color development was performed with NBT/BCIP solution (Sigma-Aldrich) overnight, then samples were washed using PBS^67^.

For IHC as a second staining after ISH, endogenous peroxidase was blocked with 0.3% H_2_O_2_ in PBS for 30 min, then sections were incubated with G-Block (Genostaff) and Avidin/Biotin Blocking Kit (Vector Laboratories). The sections were incubated with 2 µg/mL of anti-Fibroblasts rat monoclonal antibody (ER-TR7, LifeSpan BioSiences) at 2–8 °C overnight. They were incubated with biotin-conjugated rabbit anti-rat IgG (Vector), for 30 min at RT, followed by the addition of peroxidase conjugated streptavidin (Nichirei) for 5 min. Peroxidase activity was visualized by diaminobenzidine and then washed with tap-water. Then, the sections were mounted with G-Mount (Genostaff).

### Skin fibroblast isolation

In order to obtain murine skin cells, skin was collected from the back and cut into small pieces and treated with 0.1% collagenase (Wako, 032-22364) as described previously^68^. Following the collagenase treatment, cells were filtered through 70μm cell strainer (BD Biosciences, 352350) and washed with 2% NCS (Gibco, 15070063) containing RPMI1640 (nacalai tesque, 30264-56) medium. Cell sorting was conducted with FACS Aria III. Collected cells were first stained with Fc-block (BD Biosciences, 553141). Cells were then washed and stained with anti-mouse antibodies and 7-AAD. The staining patterns used for skin fibroblasts were CD90.2^+^ gp38^+^ CD45^neg^.

### Real-time quantitative RT-PCR analysis

Cells were homogenized with Sepasol RNAI (nacalai tesque, 09379-55,), and the total RNA was isolated following the manufacturer’s instructions. For standard RT-PCR, cDNA was synthesized from 500 ng of total RNA with reverse transcriptase (TRT-101, ReverTra Ace; Toyobo) and 500 ng of oligo (dT) primer (Invitrogen) for 30 min at 42 °C. A portion of the cDNA (typically 1/20 volume) was used for standard PCR to detect *Il*-*6*, *Tnf*α, *Tgf*β1 and *G3pdh*. Twenty-five cycles of PCR were performed with 0.5 U of rTaq DNA polymerase and 10 pmol of gene-specific sense and antisense primers. For real-time quantitative RT-PCR, the *Il*-*6* or *Tnf*α gene expression was measured relative to *G3pdh* using the SYBR® Green reagent (RR820A, TaKaRa)^18,69^. The primers used in these experiments were purchased from TaKaRa, and the sequences are as follows: IL-6: forward primer, 5′- GAGGATACCACTCCCAACAGACC-3′ and reverse primer, 5′-AAGTGCATCATCGTTGTTCATACA-3′; TNFα: forward primer, 5′-CATCTTCTCAAAATTCGAGTGACAA-3′ and reverse primer, 5′-TGGGAGTAGACAAGGTACAACCC-3′; TGFβ1: forward primer, 5′-GTGTGGAGCAACATGTGGAACTCTA-3′ and reverse primer, 5′-CGCTGAATCGAAAGCCCTGTA-3′.G3PDH: forward primer, 5′-TTCACCACCATGGAGAAGGCCG-3′ and reverse primer, 5′-GGCATGGACTGTGGTCATGA-3′.

### Luciferase reporter assay

The pGL4.21/human *Il*-*6* promoter (−1178/+13) vector and pRL-TK vector were transiently cotransfected into Raw264.7 cells using the Lipofectamine 2000 transfection reagent (11668019, Thermo Fisher Scientific). Cells were harvested 24 h after transfection and stimulated with the indicated reagents for 5 h. The Luciferase activity of the total cell lysates was measured using the Dual-Luciferase Reporter Assay System (E1910, Promega).

### Statistical analysis

All data were analyzed with Statcel. Data were considered statistically significant when the *P* value was less than 0.05. Statistical analyses were performed using two-tailed Student’s t-test. Additional statistical analyses were performed using Tukey-Kramer multiple comparison test.

## Supplementary information

Supplemental information
