# Supplementary material for: Mast cells play role in wound healing through the ZnT2/GPR39/IL-6 axis
Source: Sci Rep. 2019 Jul 25;9:10842. doi: 10.1038/s41598-019-47132-5 (PMC6658492; doi:10.1038/s41598-019-47132-5)

Mast cells play role in wound healing through the ZnT2 / GPR39 / IL-6 axis

Keigo Nishida[1,2,]*, Aiko Hasegawa[2,7], Satoru Yamasaki[2,3], Ryota Uchida[1], Wakana Ohashi[2,8], Yosuke Kurashima[9,10,11,12,13,14,15], Jun Kunisawa[15,16], Shunsuke Kimura[17,19], Toshihiko Iwanaga[17], Hiroshi Watarai[18], Koji Hase[19,20], Hideki Ogura[21], Manabu Nakayama[22], Jun-ichi Kashiwakura[23], Yoshimichi Okayama[24], Masato Kubo[4,25], Osamu Ohara[5], Hiroshi Kiyono[10,12,13,14,16], Haruhiko Koseki[6], Masaaki Murakami[26], and Toshio Hirano[27]

1, Laboratory of Immune Regulation, Graduate School of Pharmaceutical Sciences, Suzuka University of Medical Science, 3500-3 Minamitamagaki-cho, Suzuka, Mie 513-8670, Japan
2, Laboratory for Homeostatic Network, RIKEN Center for Integrative Medical Sciences (IMS), 1-7-22 Suehiro-cho, Tsurumi, Yokohama, Kanagawa 230-0045, Japan
3, Laboratory for Immunotherapy, RIKEN Center for Integrative Medical Sciences (IMS), 1-7-22 Suehiro-cho, Tsurumi, Yokohama, Kanagawa 230-0045, Japan
4, Laboratory for Cytokine Regulation, RIKEN Center for Integrative Medical Sciences (IMS), 1-7-22 Suehiro-cho, Tsurumi, Yokohama, Kanagawa 230-0045, Japan
5, Laboratory for Integrative Genomics, RIKEN Center for Integrative Medical Sciences (IMS), 1-7-22 Suehiro-cho, Tsurumi, Yokohama, Kanagawa 230-0045, Japan
6, Laboratory for Developmental Genetics, RIKEN Center for Integrative Medical Sciences (IMS), 1-7-22 Suehiro-cho, Tsurumi, Yokohama, Kanagawa 230-0045, Japan
7, Department of Pediatrics, Shinshu University School of Medicine, 3-1-1 Asahi, Matsumoto, Nagano 390-8621,Japan
8, Department of Molecular and Medical Pharmacology, Graduate School of Medicine and Pharmaceutical Sciences for Research, University of Toyama, Toyama, 930-0194, Japan

9, Department of Innovative Medicine, Graduate School of Medicine, Chiba University, 1-8-1 Inohana, Chuo-ku, Chiba 260-8670, Japan
10, Division of Mucosal Immunology, IMSUT Distinguished Professor Unit, The Institute of Medical Science, The University of Tokyo, 4-6-1 Shirokanedai, Minato-ku, Tokyo, 108-8639 Japan
11, Department of Mucosal Immunology, Graduate School of Medicine, Chiba University, 1-8-1 Inohana, Chuo-ku, Chiba 260-8670, Japan
12, Division of Clinical Vaccinology, International Research and Development Center for Mucosal Vaccine, the Institute of Medical Science, The University of Tokyo, 4-6-1 Shirokanedai, Minato-ku, Tokyo, Japan
13, Institute for Global Prominent Research, Chiba University, 1-8-1 Inohana, Chuo-ku, Chiba 260-8670, Japan
14, Chiba University-UC San Diego Center for Mucosal Immunology, Allergy and Vaccines (CU-UCSD cMAV), University of California San Diego, 9500 Gilman Dr. MC 0063, San Diego, CA, 92093-0063, United States
15, Laboratory of Vaccine Materials, Center for Vaccine and Adjuvant Research, and Laboratory of Gut Environmental System, National Institutes of Biomedical Innovation, Health and Nutrition (NIBIOHN), 7-6-8 Asagi Saito, Ibaraki, Osaka 567-0085, Japan
16, Division of Mucosal Vaccines, International Research and Development Center for Mucosal Vaccines, The Institute of Medical Science, The University of Tokyo, 4-6-1 Shirokanedai, Minato-ku, Tokyo 108-8639, Japan
17, Laboratory of Histology and Cytology, Department of Anatomy, Hokkaido University Graduate School of Medicine, Sapporo 060-8638, Japan
18, Department of Immunology and Stem Cell Biology, Faculty of Medicine, Institute of Medical, Pharmaceutical and Health Sciences, Kanazawa University, 13-1 Takaramachi, Kanazawa, Ishikawa 920-8640, Japan
19, Division of Biochemistry, Faculty of Pharmacy, Keio University, Tokyo 105-0011, Japan
20, Division of Mucosal Barriology, International Research and Development Center for Mucosal Vaccines, The Institute of Medical Science, The University of Tokyo (IMSUT), Minato-ku, Tokyo 108-8639, Japan

21, Department of Microbiology, Hyogo College of Medicine 1-1, Mukogawa-cho, Nishinomiya 663-8501, Japan
22, Laboratory of Medical Omics Research, Department of Frontier Research and Development, Kazusa DNA Research Institute,2-6-7 Kazusa-Kamatari, Kisarazu, Chiba 292-0818, Japan
23, Laboratory of Immunology, Graduate School of Pharmaceutical Sciences, Hokkaido University, Sapporo 060-0812, Japan
24, Allergy and Immunology Project Team, Center for Allergy, Center for Medical Education, Nihon University School of Medicine 30-1 Oyaguchi Kamicho Itabashi-Ku Tokyo 173-8610 Japan
25, Division of Molecular Pathology, Research Institute for Biomedical Science, Tokyo University of Science, 2669 Yamazaki, Noda-shi, Chiba 278-0022, Japan
26, Division of Molecular Psychoimmunology, Institute for Genetic Medicine and Graduate School of Medicine, Hokkaido University, Sapporo 060-815, Japan.
27, Headquarters, National Institutes for Quantum and Radiological Science and Technology, 4-9-1 Anagawa, Inage-ku, Chiba, 263-8555, Japan

*Corresponding author: Keigo Nishida

Keigo Nishida, PhD, Laboratory of Immune Regulation, Graduate School of Pharmaceutical Sciences, Suzuka University of Medical Science, 3500-3 Minamitamagaki, Suzuka, Mie 513-8607, JAPAN

Tel: +81-59-340-0577 Fax: +81-59-368-1271

E-mail: knishida@suzuka-u.ac.jp

Supplementary Figure legends

Supplementary Figure S1. Expression profile of ZnT family members in BMMCs

The expression level of ZnT family members was determined from the ReFDIC (Reference Database of Immune Cells, http://refdic.rcai.riken.jp/).

Supplementary Figure S2. Isotype-matched antibody stain images for Figure 1A.

Double immunostaining of CD63 (red) and ZnT2 (green) in mast cells. Normal Rabbit IgG is used as negative control. Scale bar: 2 μm and 10 μm.

Supplementary Figure S3. Full-length blot images for Figure 1C.

Supplementary Figure S4. Expression of ZnT2 in *ZnT2$^{+/-}$* *and ZnT2$^{-/-}$* -derived BMMC.

Immunoblotting analysis of ZnT2 protein in BMMCs using anti-ZnT2 antibody.

Supplementary Figure S5. Full-length blot images for Figure S4.

Supplementary Figure S6. Normal development of *ZnT2$^{-/-}$* BMMCs.

(A) Histological analysis of mast cells in the ear of *ZnT2$^{+/-}$* (n = 3) and *ZnT2$^{-/-}$* (n = 3) mice. Sections were stained with nuclear fast red and Alcian blue. The arrows

indicate mast cells. (B) Representative flow cytometry dot plot profiles of freshly isolated peritoneal cells from *ZnT2*$^{+/-}$ (left) and *ZnT2*$^{-/-}$ (right) mice stained for CD117 and FcεRI. (C) Flow cytometric analysis of BMMCs from *ZnT2*$^{+/+}$ and *ZnT2*$^{-/-}$ mice. c-Kit and FcεRI expression was detected by staining with anti-CD117 (c-kit) and anti-IgE, respectively.

Supplementary Figure S7. Normal production of cytokines and β–hexosaminidase by *ZnT2*$^{-/-}$ BMMCs.

(A) BMMC degranulation assessed by β–hexosaminidase release. BMMCs from *ZnT2*$^{+/+}$ and *ZnT2*$^{-/-}$ mice were sensitized with anti-DNP IgE and stimulated with DNP–HSA. As a control, BMMCs were stimulated with 1 μM ionomycin (Io). Values represent the mean + SD. (B) Enzyme-linked immunosorbent assays for IL-6 and TNFα produced by BMMCs from *ZnT2*$^{+/+}$ and *ZnT2*$^{-/-}$ mice sensitized with anti-DNP IgE and stimulated with DNP–HSA. Values represent the mean + SD. (c) LPS-induced cytokine production by *ZnT2*$^{+/+}$ and *ZnT2*$^{-/-}$ BMMCs stimulated with 1 μg/ml LPS for the indicated time periods. Values represent the mean + SD.

Supplementary Figure S8. Mast cells are required for normal wound healing.

(A) Impaired wound closure in *Kit*$^{W-sh}$/*Kit*$^{W-sh}$ (mast cell-deficient) mice (n = 7 animals) was prevented by prior reconstitution of the dermis with control BMMCs (n = 10 animals). Values represent the mean + SEM; *P < 0.05

comparing *Kit*$^{W-sh}$/*Kit*$^{W-sh}$ and C57BL/6 mice (n = 8 animals); +P < 0.05 comparing *Kit*$^{W-sh}$/*Kit*$^{W-sh}$ mice and mast cell-reconstituted *Kit*$^{W-sh}$/*Kit*$^{W-sh}$ mice (BMMCs→ *Kit*$^{W-sh}$/*Kit*$^{W-sh}$ mice). (B) Mas-TRECK Tg mice and C57BL/6 mice were subjected to wound healing analysis. For diphtheria toxin (DT) treatment, mice were injected with 250 ng of DT intraperitoneally for 5 consecutive days (black arrows). The wound area (%) is shown as 100 % on Day 0 value and the values are expressed as means±s.e.m. (n=5 for Tg; n=5 for C57BL6), **P **<** 0.01 (two-tailed Student's t-test).

Supplementary Figure S9. Numbers of mast cells in dermis from back skin.
Absolute numbers of mast cells expressed as number per mm^2 of back skin between *ZnT2*$^{+/+}$ (n = 16) and *ZnT2*$^{-/-}$ (n = 13) BMMCs-engrafted *Kit*$^{W-sh/W-sh}$ mice. Data show mean + s.d.

Supplementary Figure S10. Full-length blot images for Figure 5B.

Supplementary Figure S11. Full-length blot images for Figure 5D.

Supplementary Figure S12. Schematic diagram of the human *Il-6* promoter constructs.
The indicated *Il-6* promoter-derived recombinant reporter gene constructs were used in Figure 5F. Boxes, transcription factor-binding sites; crossed boxes,

transcription factor-binding sites of the human *Il-6* promoter (-1178/+13) with point mutations.

Supplementary Figure S13. *In situ* hybridization analysis of skin wound samples from WT mice 3 days after injury.

Skin samples were hybridized with a *Gpr39*-specific probe. Left panel shows unwounded skin. Right panel shows wounded skin. The arrows indicate *gpr39* positive cells.

Supplementary Figure S14. Proposed mechanism by which Zn functions as an inflammatory mediator.

A mechanism showing that Zn is a novel mediator of the inflammatory response. Upon injury, endogenous inflammatory mediators or "danger signals" are released and activate mast cells, which then release Zn into the extracellular space. In addition to this, it is well known that keratinocytes release Zn during injury. Released Zn directly binds to the Zn receptor GPR39 on skin fibroblast and immune-related cells and induces the expression of cytokines such as IL-6 and TNFα. These cytokines then contribute to inflammatory responses such as wound healing.

Supplemental fig.1.

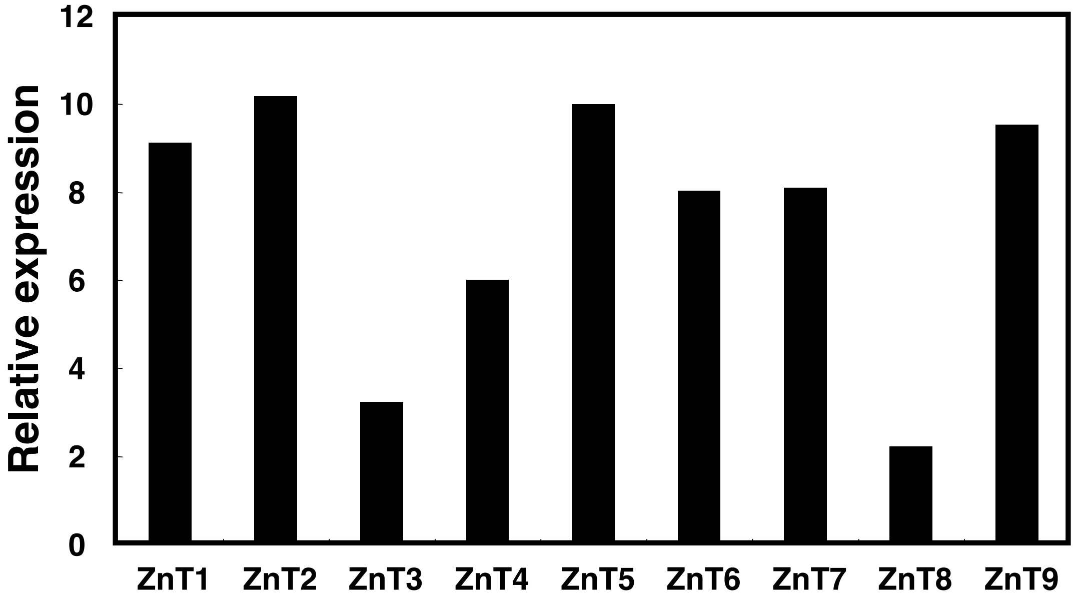

Supplemental fig.2. Isotype-matched antibody stain images for Figure 1A.

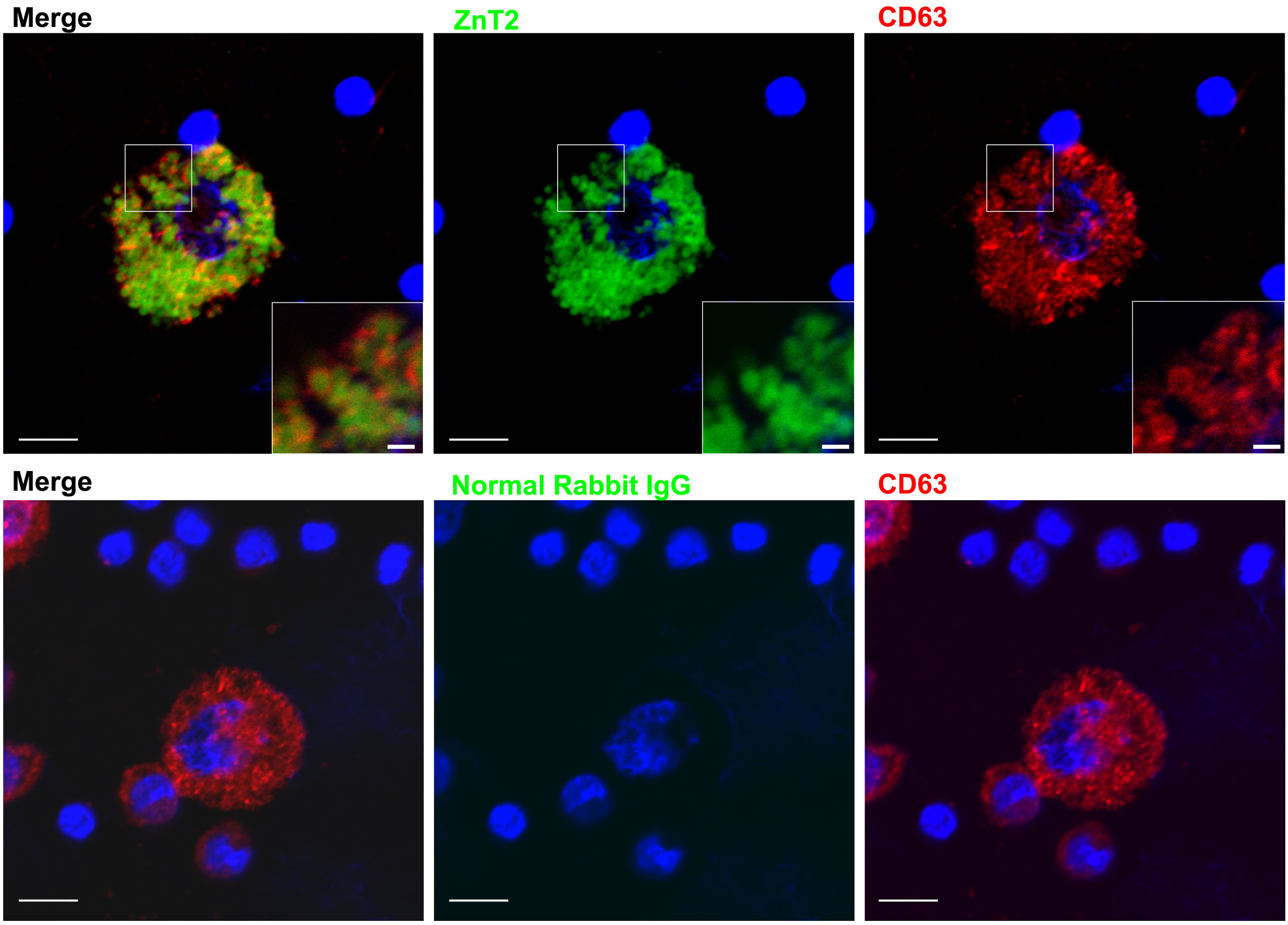

Supplemental fig.3. Full-length blot images for Figure 1C.

Fraction number

1 3 5 7 9 11 13 15 17 19 21 23 25 27 29 31

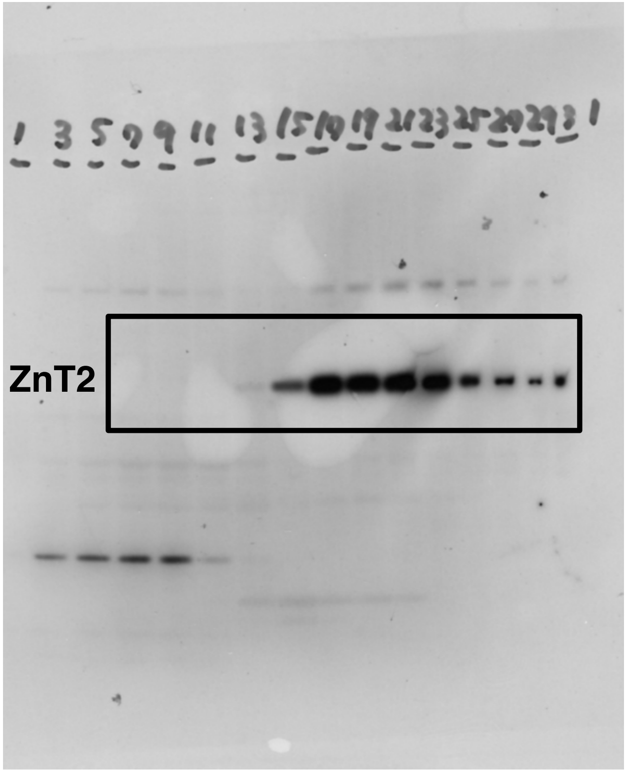

Full unedited gel for Figure 1C upper

Fraction number

1 3 5 7 9 11 13 15 17 19 21 23 25 27 29 31

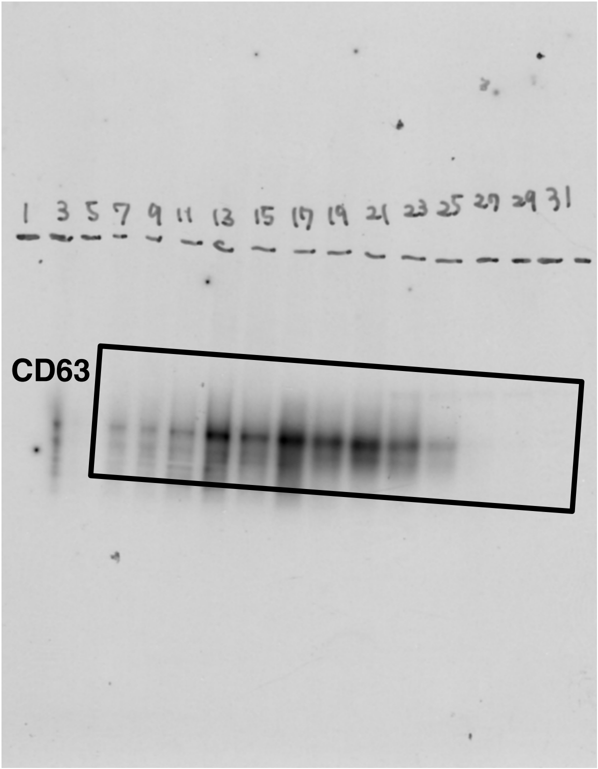

Full unedited gel for Figure 1C lower

Supplemental fig.4.

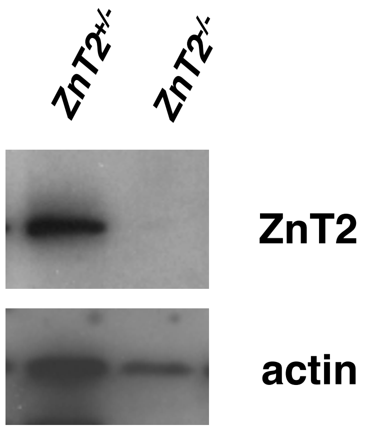

Supplemental fig.5. Full-length blot images for Figure S4.

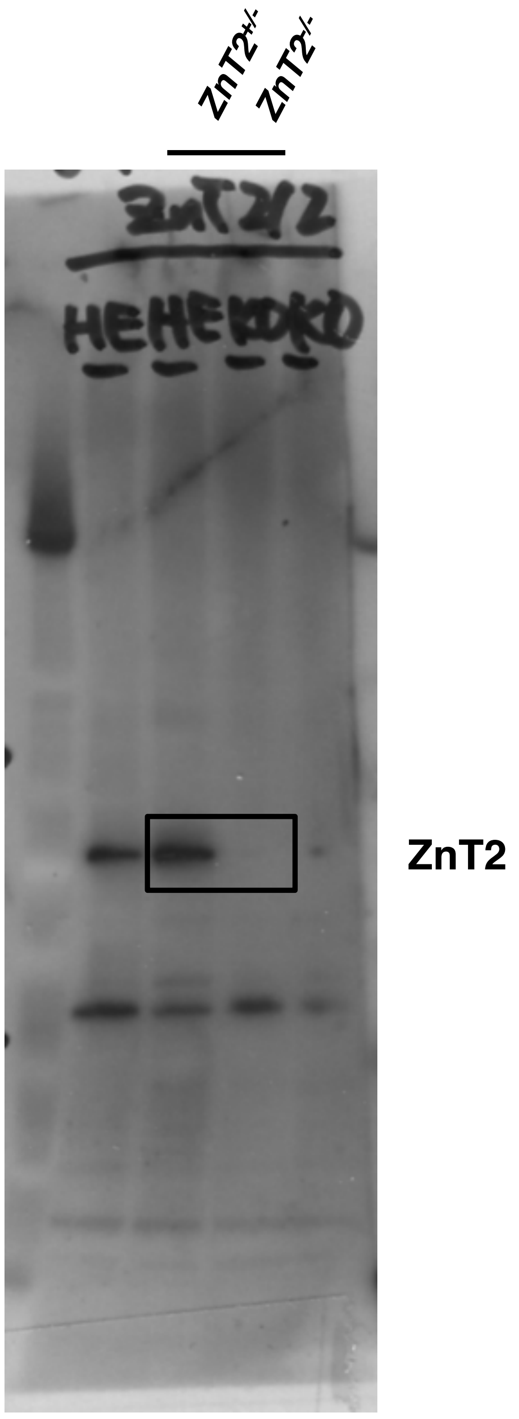

Full unedited gel for Figure S2B upper

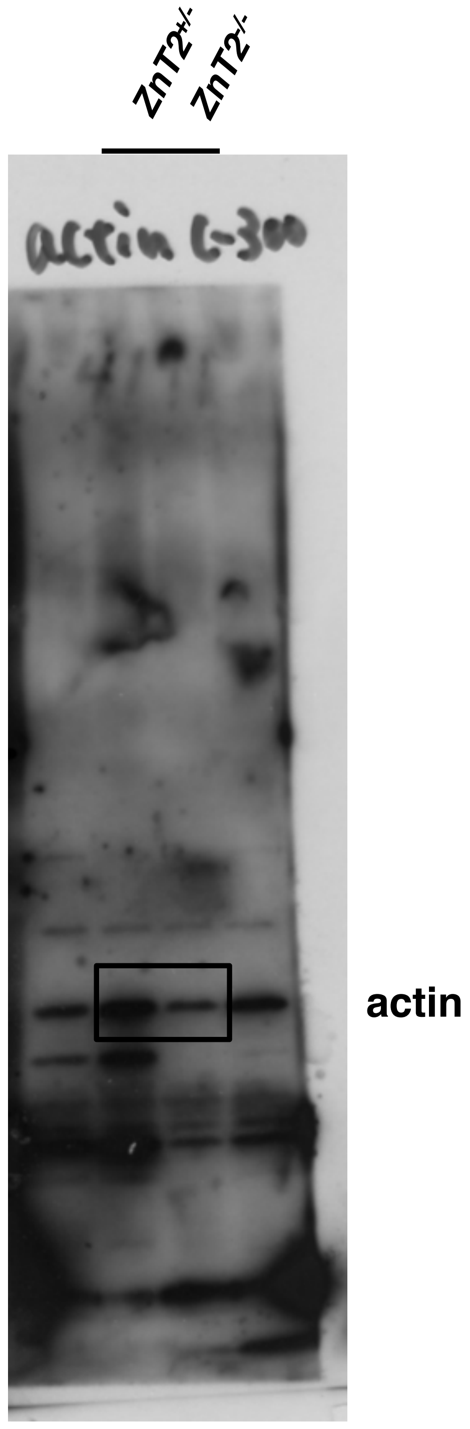

Full unedited gel for Figure S2B lower

Supplemental fig.6.

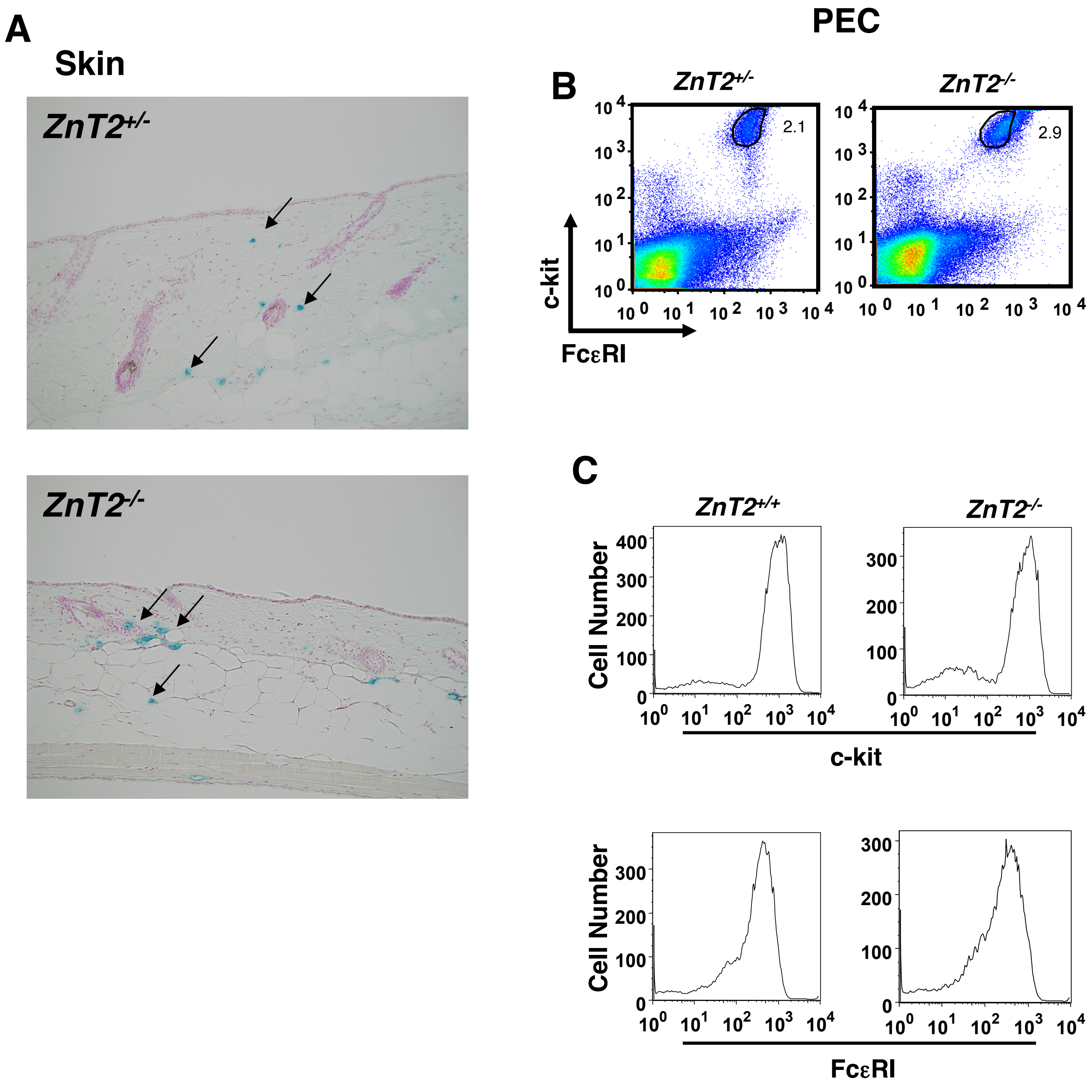

Supplemental fig.7.

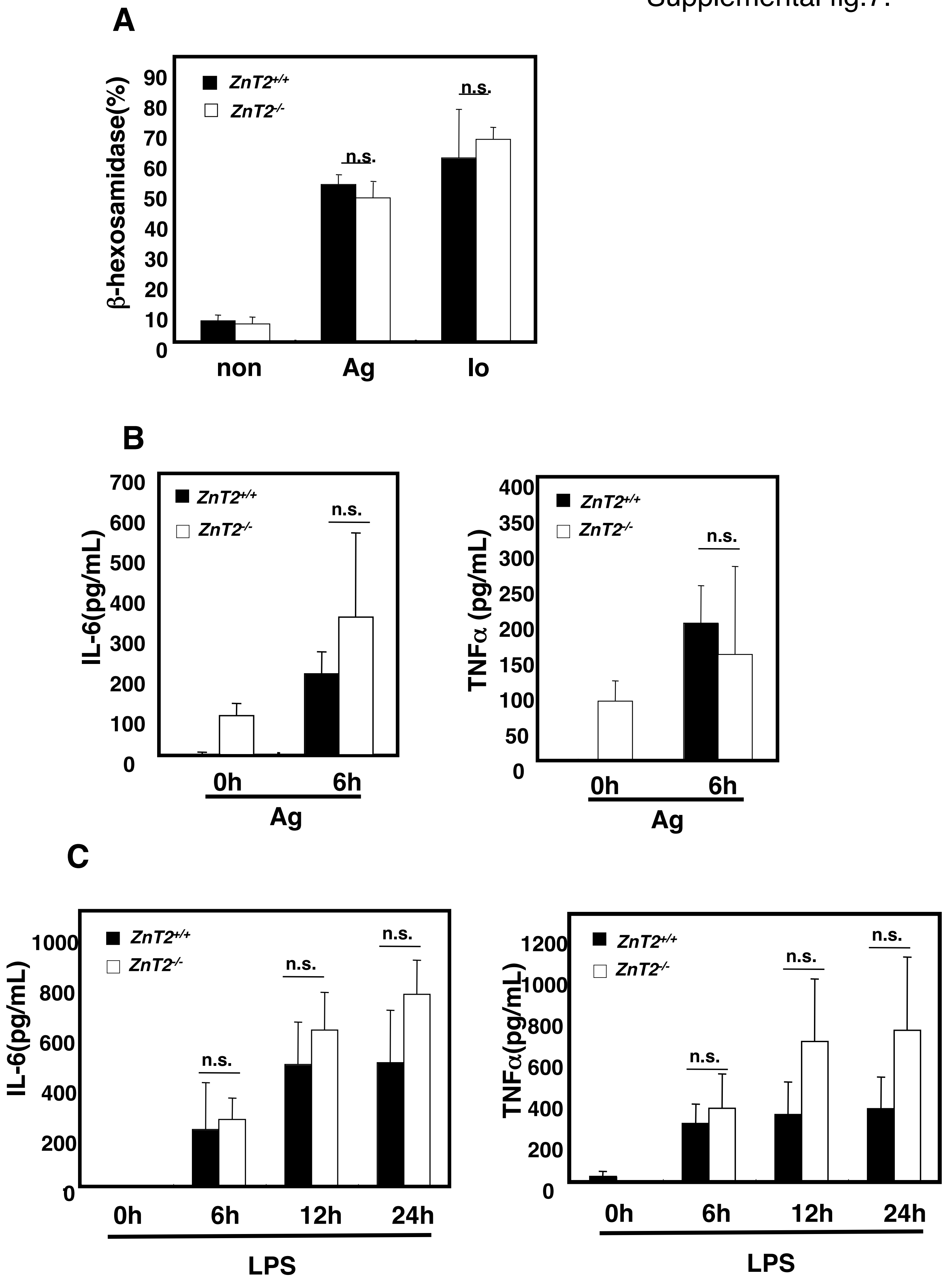

Supplemental fig.8.

A

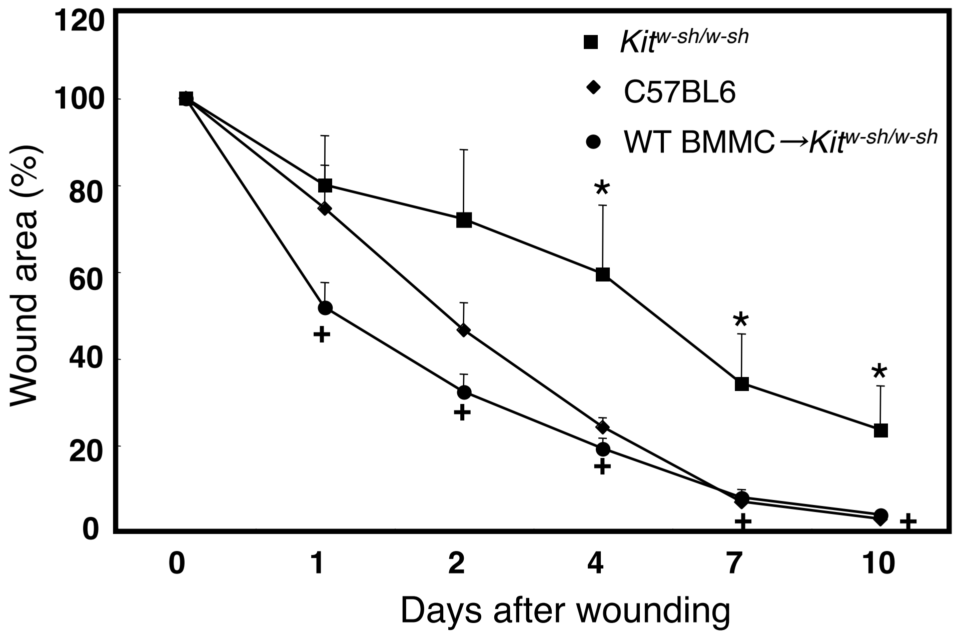

B

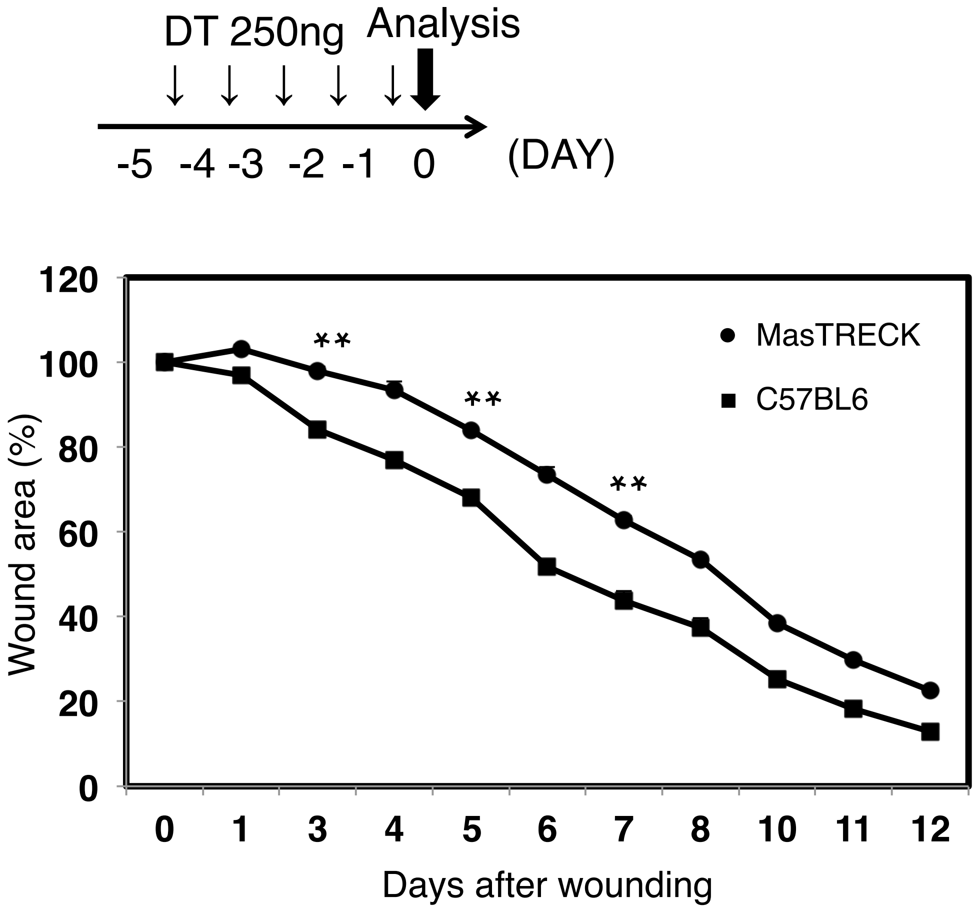

Supplemental fig.9.

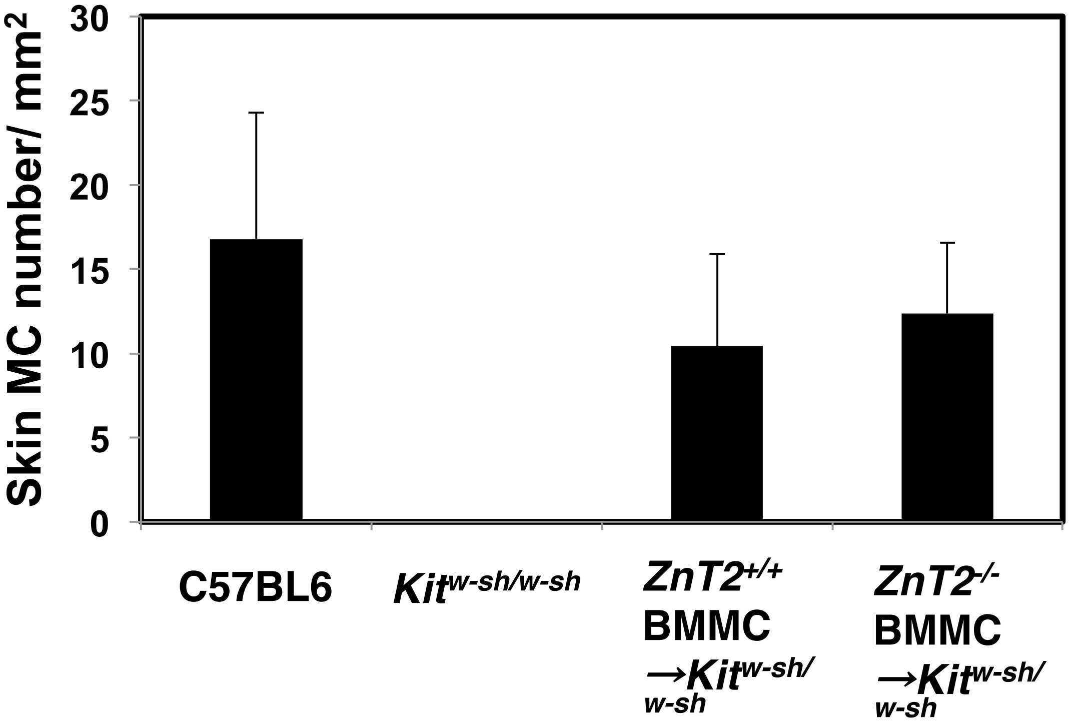

Supplemental fig.10. Full-length blot images for Figure 5B.

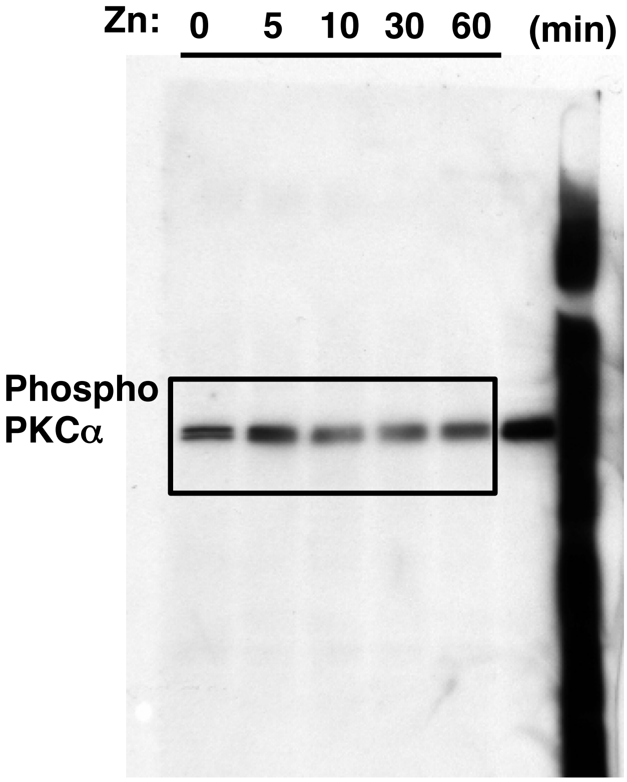

Full unedited gel for Figure 5B upper

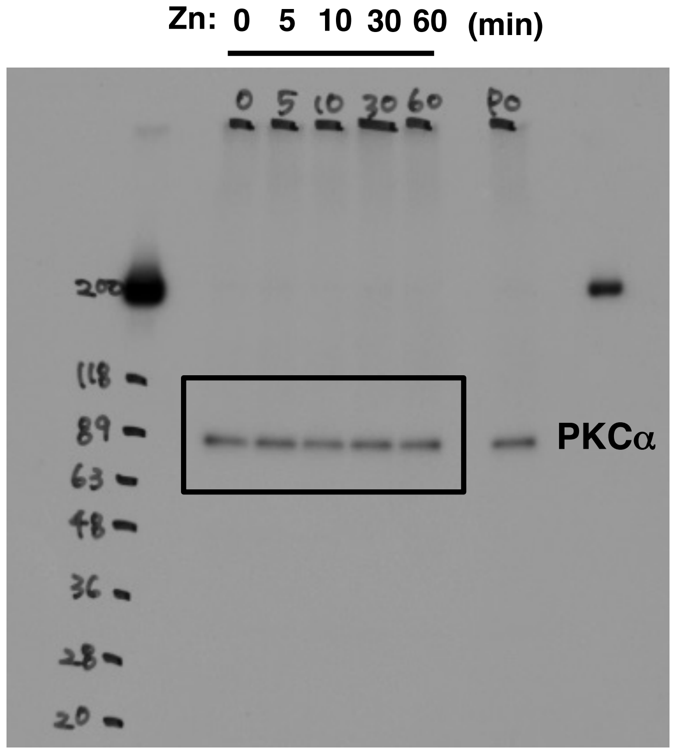

Full unedited gel for Figure 5B lower

Supplemental fig.11. Full-length blot images for Figure 5D.

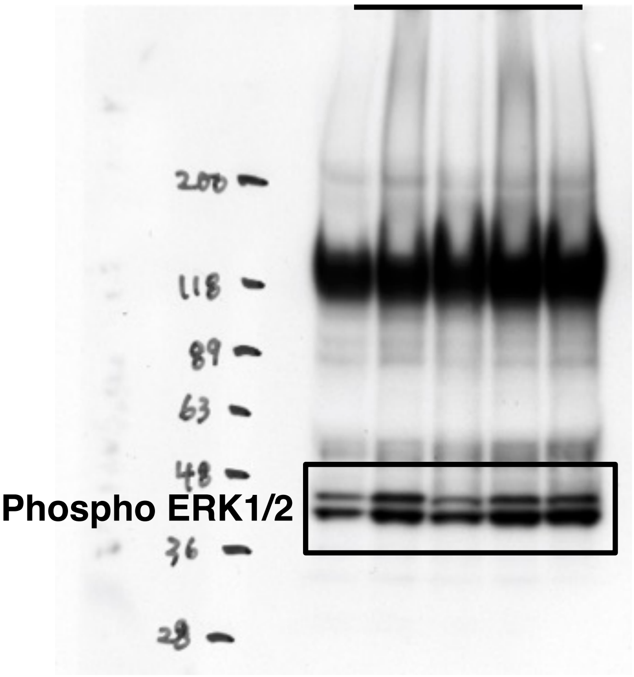

Full unedited gel for Figure 5D upper

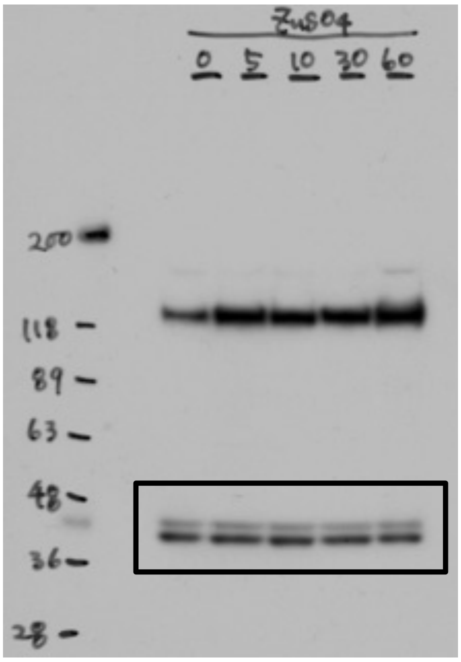

Full unedited gel for Figure 5D lower

Supplemental fig.12.

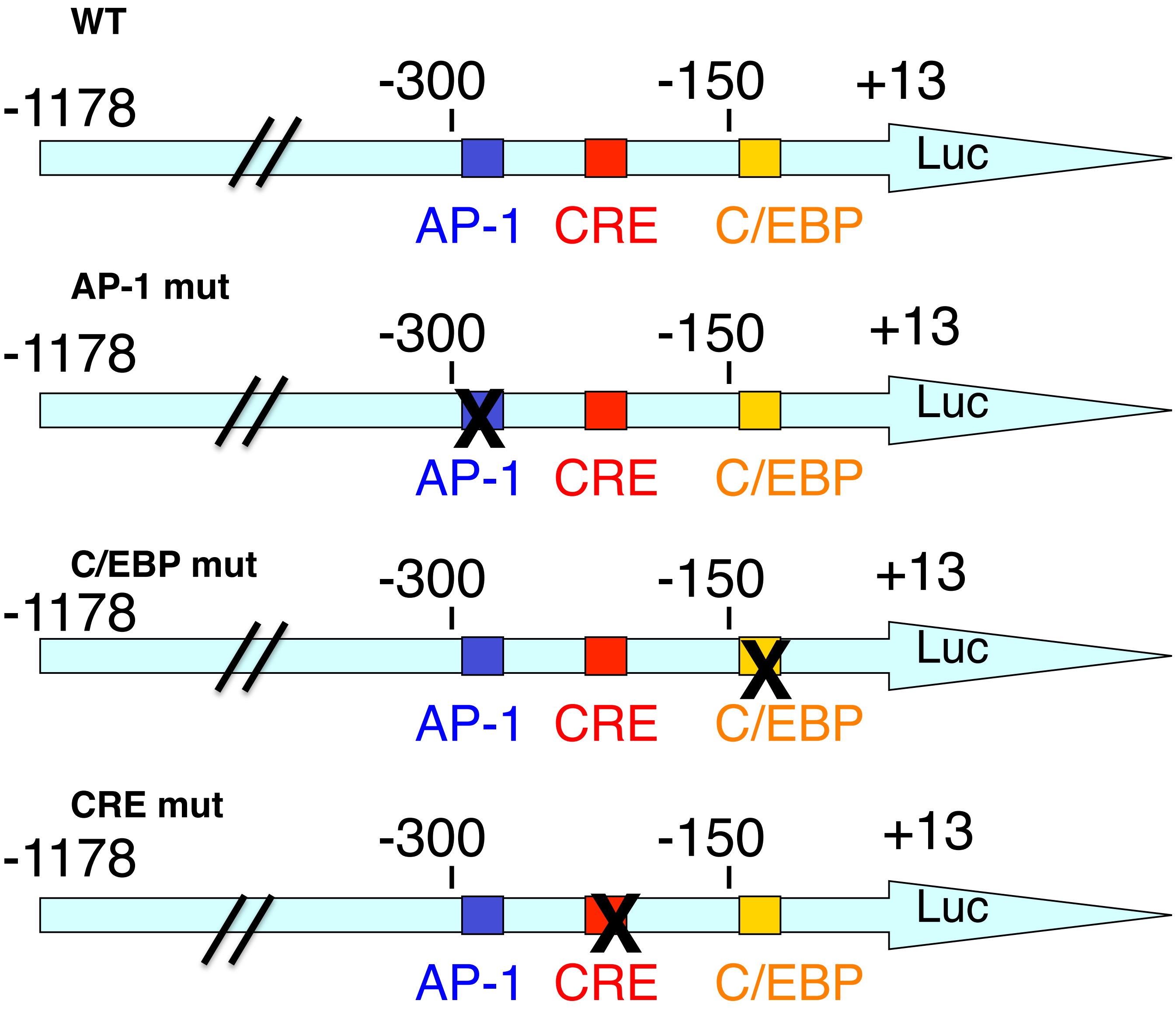

Supplemental fig.13.

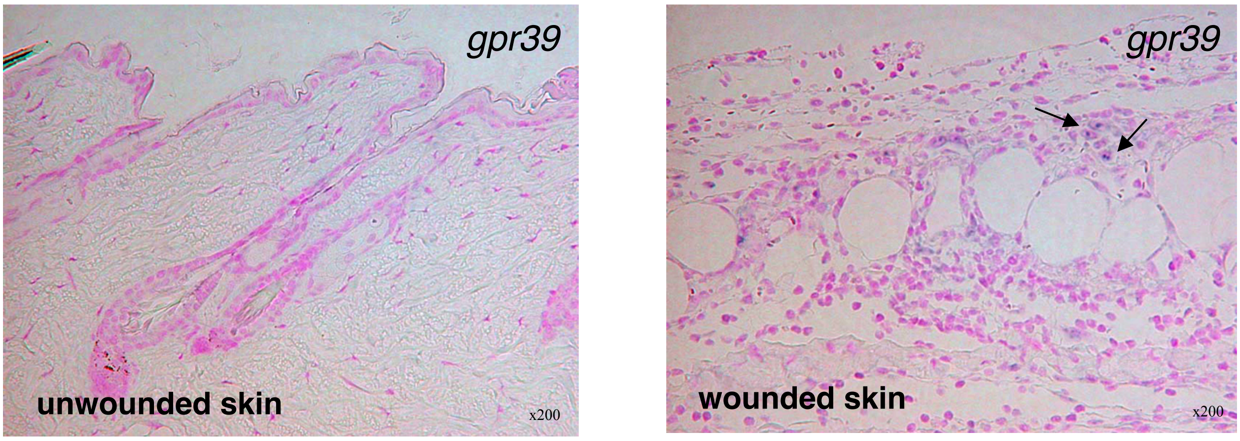

Supplemental fig.14.

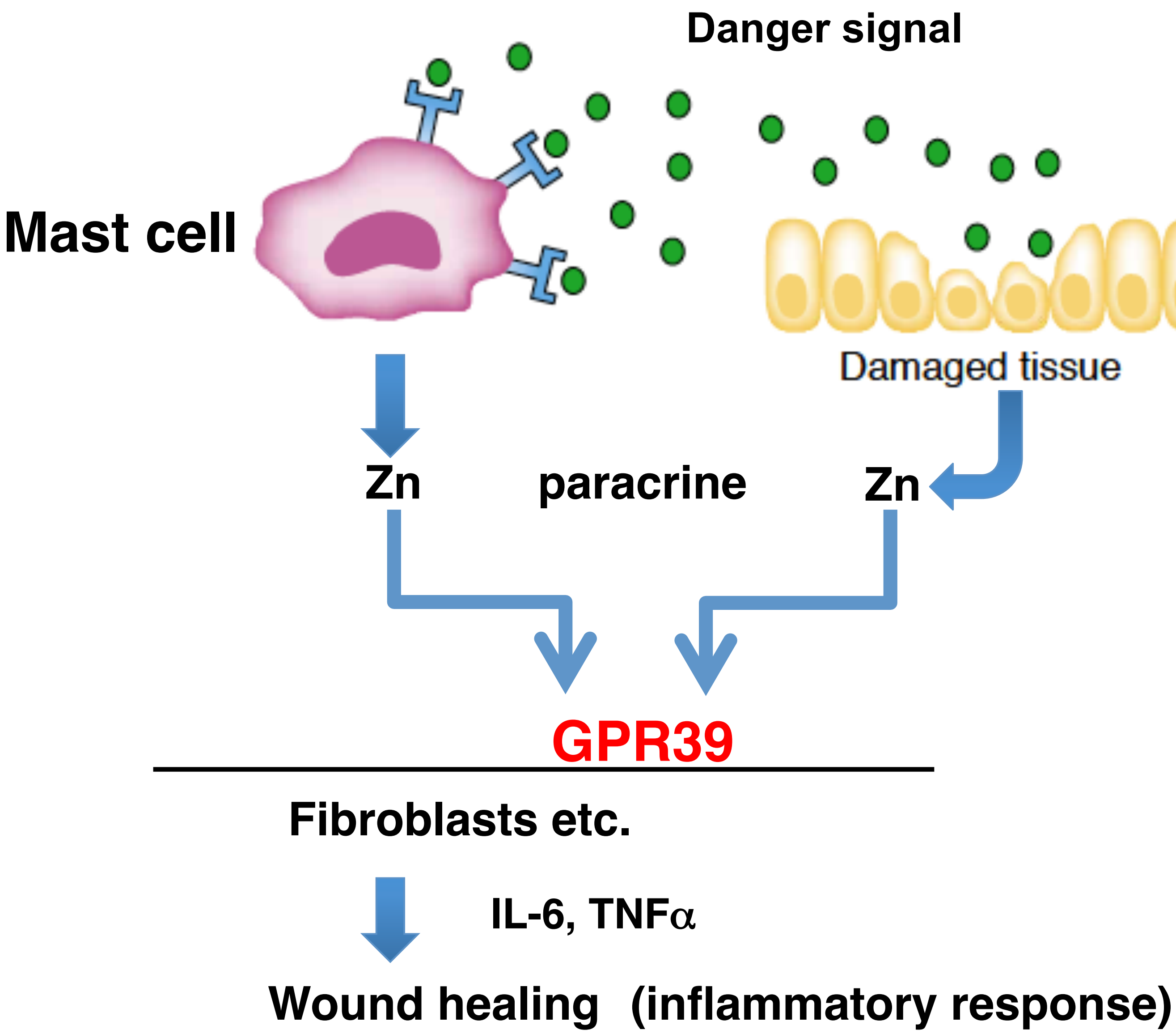

Supplement: Supplementary file 1 — Supplemental information [file 41598_2019_47132_MOESM1_ESM.pdf]
